# DNA polymerase kappa stabilized by Ptbp2 interacts with MRE11 and promotes genomic instability in leukemia

**DOI:** 10.1038/s41420-026-02951-0

**Published:** 2026-02-10

**Authors:** Shristi Lama, Bibhudev Barik, Sajitha IS, Tannistha Sarkar, Sayantan Chanda, Monalisa Behera, Payel Guha, Subhankar Priyadarshi Behera, Sutapa Biswas, Sonali Mohapatra, Ghanashyam Biswas, Soumen Chakraborty

**Affiliations:** 1 Cancer Biology Group, Institute of Life Sciences, Nalco Square, Bhubaneswar, India; 2 Regional Centre for Biotechnology, Faridabad, India; 3 Department of Veterinary Pathology, Kerala Veterinary & Animal Sciences University, Wayanad, Kerala India; 4 Sparsh Hospital and Critical Care, Bhubaneswar, India; 5 Department of Medical Oncology/Haematology, All India Institute of Medical Sciences, Bhubaneswar, India

**Keywords:** Chronic myeloid leukaemia, Oncogenes

## Abstract

Polypyrimidine Tract Binding Protein 2 (Ptbp2) binds to polypyrimidine clusters in pre-mRNA molecules and plays a vital role in alternative splicing, especially during neuronal development and maturation. Our study shows that Ptbp2 binds to the 3’ UTR of DNA polymerase kappa (Polk), leading to its stabilization and increased expression. While Polk’s role in DNA repair is known, its post-transcriptional regulation remains largely unclear. We observed a correlation between increased Ptbp2 levels and higher Polk expression in clinical samples of Chronic Myeloid Leukemia (CML). Knocking out Ptbp2 in CML cell lines and patient samples decreased Polk levels; when treated with hydroxyurea, these samples exhibited increased DNA damage, evidenced by long comet tails and elevated γH2AX foci, a DNA damage marker; however, re-expressing Polk in Ptbp2-KO cells restored the phenotype. Disruption of the DNA repair pathway is a hallmark of cancer and is closely linked to genomic instability. Polk was found to interact with MRE11 of the MRN complex, regulating the activation of the ATM-CHK2 signaling pathway. Cells with high levels of Ptbp2 and Polk showed increased sister chromatid exchanges and BrdU incorporation in ex vivo tests, while multinucleated cells with multipolar spindles appeared in in vivo tests. Our results confirm the key role of the Ptbp2-Polk-MRE11 axis in promoting genomic instability and supporting the survival of cells with higher malignancy.

## Introduction

CML, a myeloproliferative disorder, is characterized by the causative fusion of the Philadelphia chromosome, t (9;22) (q34; q11.2), which results in the BCR::ABL1 chimeric gene [1]. This fusion leads to constitutively activated tyrosine kinase activity, triggering several associated downstream pathways, underscoring the severity of the disease [1]. Tyrosine kinase inhibitors have been reported to provide excellent clinical benefits for the past 20 years; however, this has not been observed in the blast crisis (BC) phase of the disease [2–4]. Earlier, we showed that in the BC phase of CML, BCR::ABL1 enhances the functional expression of Ptbp2, an RNA-binding protein that promotes cell proliferation and tumor formation, and enhances autophagy through Bnip3, thereby supporting its role as an oncogene in CML [5, 6]. BCR::ABL1 also generates reactive oxygen species that cause DNA breaks, leading to defective repair and increased genomic instability, which promotes disease progression [7–10]. Regardless of whether BCR::ABL1 has a direct or an indirect role in promoting genomic instability, 60–80% of patients with CML develop additional non-random chromosomal abnormalities involving chromosomes 8, 17, 19, and 22, with duplication of the Ph chromosome or trisomy 8 being the most frequent [11]. Recent reports suggest that the increasing presence of both γH2AX and 53BP1 foci in cells indicates that the promotion of NHEJ and MMEJ serves as a critical mechanism for DNA repair during blastic transformation [12].

Experimental evidence strongly suggests that activated oncogenes trigger replication stress, leading to the stalling and collapse of DNA replication forks and the formation of DNA double-strand breaks (DSBs) that, coupled with error-prone repair processes, play a crucial role in the genomic instability seen in most human cancers. DNA polymerase kappa (Polk) contributes to the replication checkpoint response and is required for recovery after replication stress. Overexpression of Polk has been reported to contribute to aneuploidy and genetic heterozygosity, thereby causing genomic instability in different carcinomas [13, 14]. The overexpression of Polk has been associated with temozolomide resistance, and its inhibition markedly sensitized cells to temozolomide by disrupting HR-mediated repair and ATR-CHK1 activation [15].

In this study, we have unveiled the unique role of Ptbp2 in stabilizing the DNA polymerase kappa enzyme, which recruits the MRN complex via interaction with MRE11, and in stimulating downstream ATM and CHK2 signaling, fostering genomic instability and disease progression.

## Results

### PTBP2 regulates DNA polymerase kappa in CML cells

**Fig. 1:**
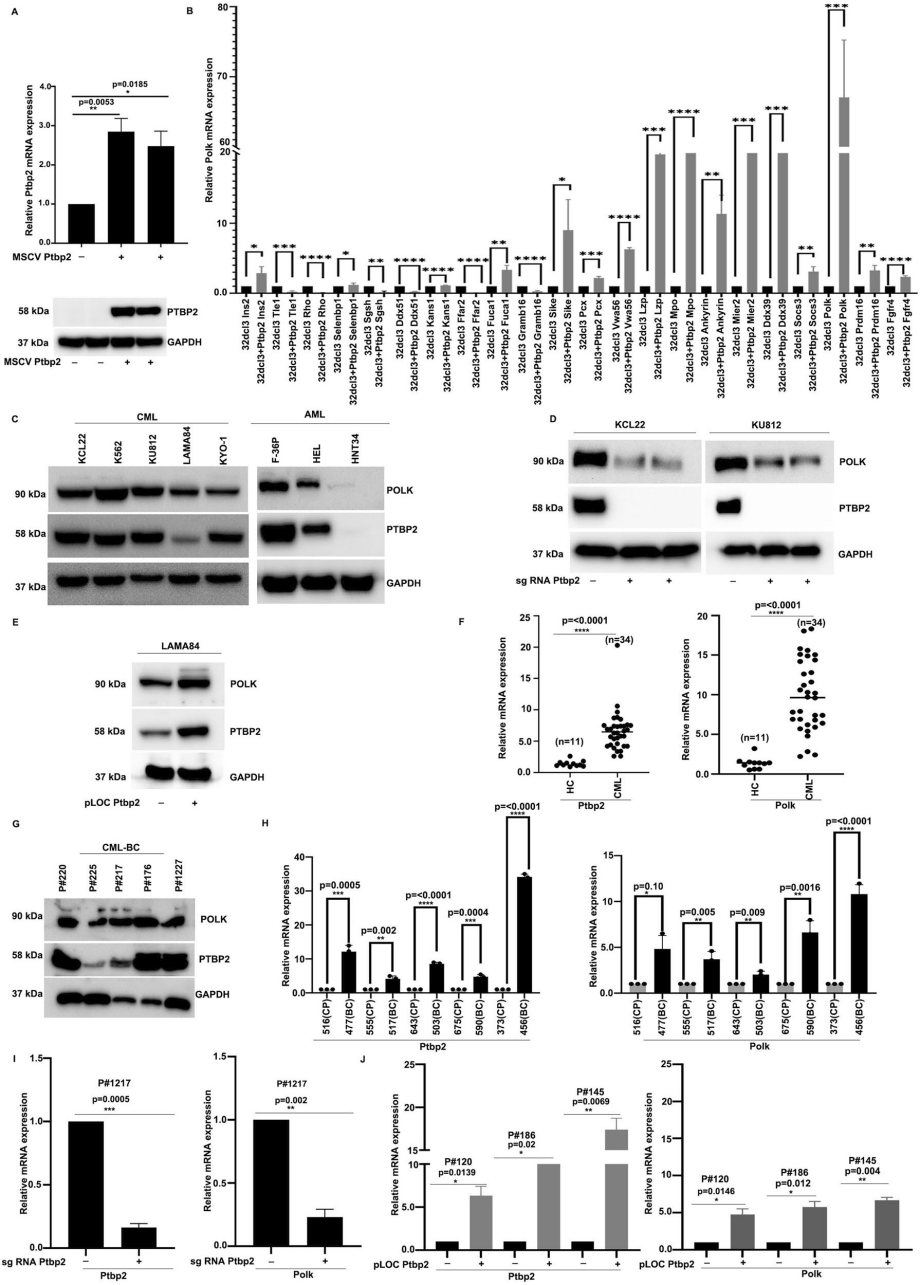
PTBP2 facilitates the upregulation of DNA polymerase kappa. **A** mRNA and protein expression of Ptbp2 in vector-32Dcl3 and 32Dcl3-Ptbp2 cells. **B** Differential expression of the targets by RT-qPCR. **C** Western blot analysis for Polk, Ptbp2, and Gapdh in different CML and AML cell lines. **D** Western blot analysis of Polk (upper panel) and Ptbp2 (middle panel) in WT NTC cells and two different clones of Ptbp2 KO in each of the KCL22 and KU812 cells. Gapdh was used as a loading control. **E** Expression of Polk in Ptbp2 overexpressing LAMA84 cells. **F** Expression of Polk and Ptbp2 in healthy control (HC) and CML patients. **G** Protein expression of Ptbp2 and Polk in 5 CML BC patients. **H** mRNA expression of Ptbp2 and Polk in 5 CML paired patients. **I** Genetic ablation of Ptbp2 in CML BC sample p#1217, followed by mRNA expression of Ptbp2 and Polk, respectively. **J** Overexpression of Ptbp2 in the CML CP samples, followed by the mRNA expression of Ptbp2 and Polk.

To identify Ptbp2 targets in hematopoietic cells, we overexpressed Ptbp2 in the 32Dcl3 murine myeloid progenitor cell line. Ptbp2 expression in these cells was confirmed by RT-qPCR and western blot analysis (Fig. 1A, top and bottom). Gene expression analysis of vector- 32Dcl3 and Ptbp2-32Dcl3 cells revealed statistically significant genes, represented in the heatmap (Supplementary Fig. 1A). Notably, the expression of Polk gene, was markedly elevated in presence of Ptbp2 (Fig. 1B). Consistent with our earlier reports, Ptbp2 expression was found to be uniform across all tested CML and AML cell lines [6], with Polk expression mirroring that of Ptbp2 (Fig. 1C). As previously noted, we utilized Ptbp2-KO-KCL22 and Ptbp2-KO-KU812 cells, along with NTC controls, confirming Ptbp2 knockout by RT-qPCR and western blot [6]. We observed decreased Polk expression in both Ptbp2-KO-KCL22 and Ptbp2-KO-KU812 cells compared to NTC cells, as evidenced by both mRNA (Supplementary Fig. 1B) and protein levels (Fig. 1D, upper panel). Earlier studies had also shown overexpression of Ptbp2 in the CML cell line LAMA84 [6]. In our analysis, we found that Polk was upregulated in Ptbp2-OE-LAMA84 cells compared to vector control at both mRNA and protein levels (Supplementary Fig. 1C, E). In 34 CML samples and 11 healthy controls, Ptbp2 and Polk expression were significantly higher in CML. A positive protein-level correlation was observed in multiple CML-BC samples (P#176, P#217, P#220, P#225, P#1227). Representative densitometry is shown (Fig. 1G and Supplementary Fig. 1D). Significantly, both Ptbp2 and Polk were upregulated in paired CML cases (Fig. 1H). We knocked out Ptbp2 from the BC sample P#1217 using a specific sgRNA, resulting in decreased expression of both Ptbp2 and Polk (Fig. 1I). Additionally, we overexpressed Ptbp2 using precision lentiORF Ptbp2 with a stop codon in CML CP patient samples (P#120, P#186, P#145), leading to an increase in Polk expression (Fig. 1J). Overall, the data suggest that Ptbp2 regulates Polk in the advanced phase of the disease.

### PTBP2 targets the 3′-UTR of DNA polymerase kappa and stabilizes its expression

**Fig. 2:**
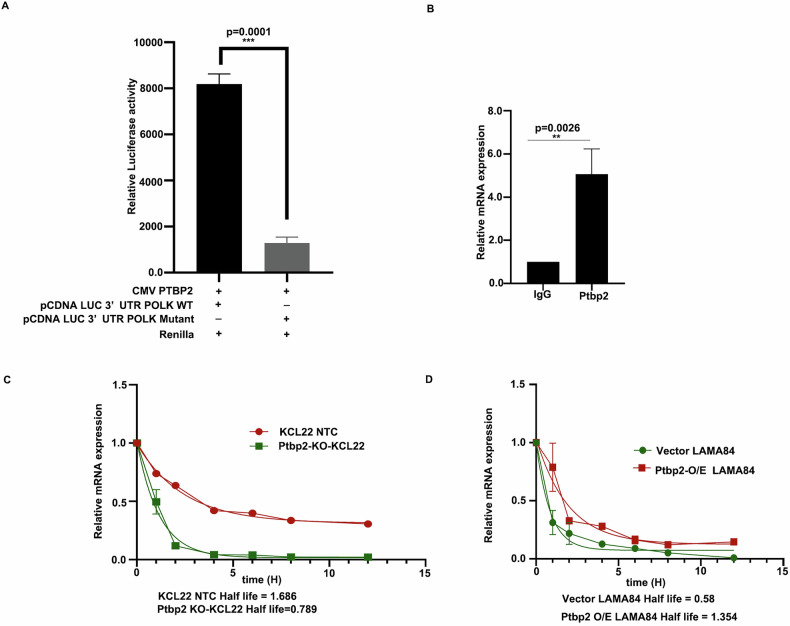
PTBP2 targets the 3′UTR of DNA polymerase kappa. **A** HEK293T cells were co-transfected with CMV Ptbp2, pcDNA LUC 3′UTR Polk WT, and pcDNA LUC 3′UTR Polk mutant. Renilla luciferase was used as an internal control, and the cells were harvested for luciferase assay after 48 h. The results were expressed as mean ± SEM from triplicate experiments. **B** RT-qPCR of Ptbp2-bound Polk transcript concerning IgG control. **C** mRNA half-life of Polk transcript in KCL22 NTC and Ptbp2-KO-KCL22 cells. **D** mRNA half-life of Polk transcript in LAMA84 and Ptbp2 O/E LAMA84 cells.

To further evaluate the mechanism by which Ptbp2 regulates Polk, we scanned the entire mRNA sequence using beRBP (https://bioinfo.vanderbilt.edu/beRBP/predict.html). Two specific PTBP2 binding sites (CUUUUCU and CUUUCU) were observed in the 3′UTR with binding scores of 0.566 and 0.492, respectively (Supplementary Fig. 2A). The PTBP2 binding sites were cloned into a luciferase vector and transfected with wild-type Ptbp2 and Renilla luciferase into HEK-293T cells. Luciferase expression increased with binding site 1 (CUUUUCU) but not site 2, and mutating site 1 (CAAAACA) reduced luciferase activity, confirming PTBP2 binds and regulates Polk stability (Fig. 2A). Again, Ptbp2 and its associated RNA were immunoprecipitated with a Ptbp2-specific antibody. The immunoprecipitated Ptbp2 was initially checked with the western blot (Supplementary Fig. 2B). The binding efficiency of Ptbp2 to the associated Polk mRNA was observed using RT-qPCR (Fig. 2B). To further validate the stabilization of Polk mRNA by Ptbp2, KCL22-NTC and Ptbp2-KO-KCL22 @ 0.5 million cells were treated with 5 μg/mL of actinomycin D, and the RNA was harvested at 0-, 5-, 10-, and 15-h post-treatment. We measured the mRNA half-life of Polk by determining transcript abundance by RT-qPCR. Half-life was 1.68 h at the Polk gene in KCL22-NTC cells and 0.789 h in Ptbp2-KO-KCL22 cells, indicating a significant decrease in the stability of Polk in the Ptbp2*-*depleted state (Fig. 2C). Overexpression of Ptbp2 in the LAMA84 cells showed a half-life of Polk of 1.35 h compared to vector-transduced LAMA84 cells, which was 0.58 h (Fig. 2D). Collectively, these data suggest that Ptbp2 binds to the 3’UTR and regulates the stability of Polk.

### The Ptbp2-polk axis acts as a regulator of DNA repair in CML

**Fig. 3:**
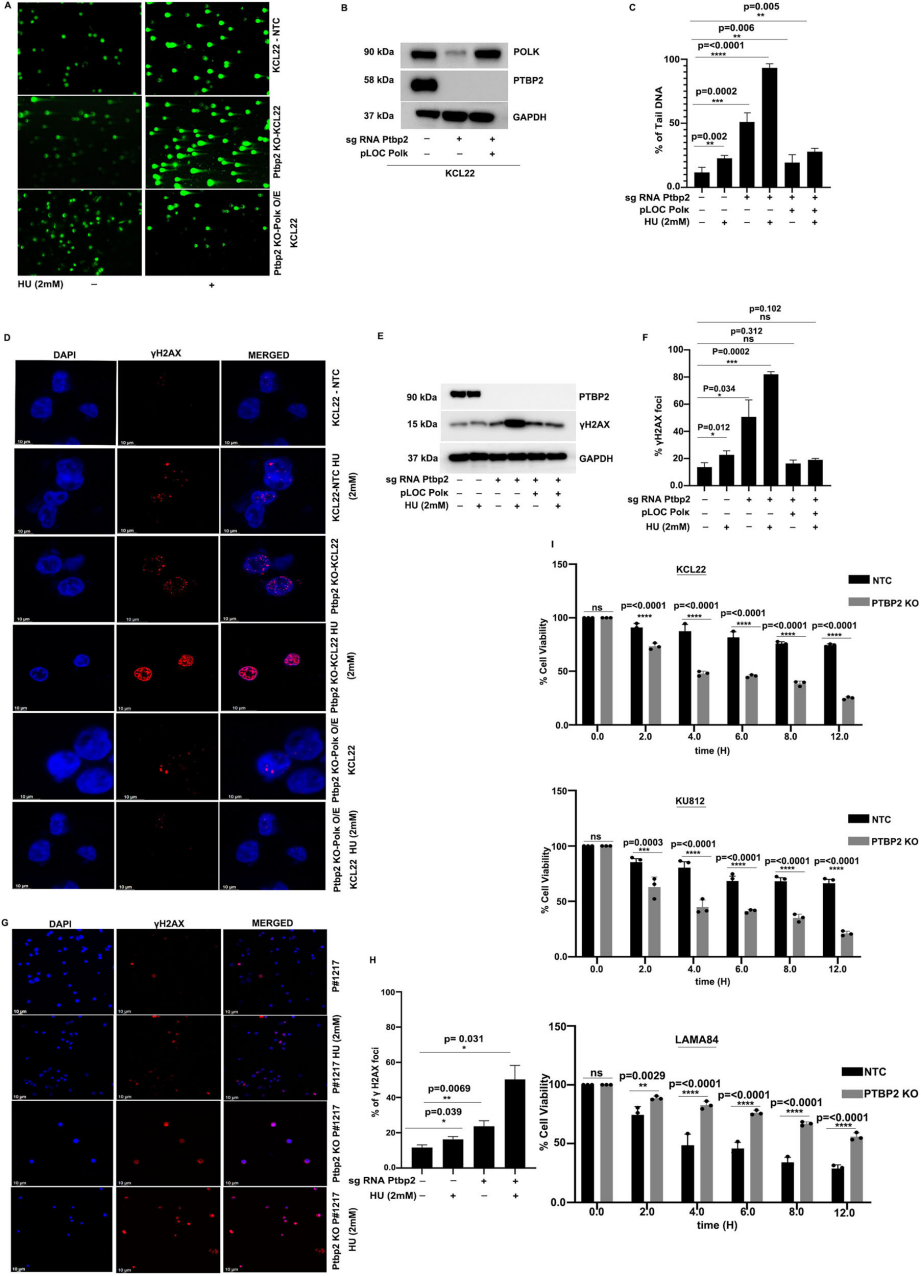
PTBP2-polk axis acts as a regulator of DNA repair. **A** KCL22-NTC, Ptbp2-KO-KCL22, and Ptbp2-KO-Polk O/E-KCL22 cells were treated with and without hydroxyurea and subjected to alkaline comet assay. **B** Represents the protein expression of the Polk in the respective cells used for the alkaline comet assay. **C** Quantification of the percentage of DNA tail. The data are presented as the mean ± SEM. **D** KCL22-NTC, Ptbp2-KO-KCL22, and Ptbp2-KO-Polk O/E-KCL22 cells treated with or without hydroxyurea and probed with γH2AX antibody and DAPI. **E** Protein expression of γH2AX and Ptbp2 in KCL22-NTC, Ptbp2-KO-KCL22, and Ptbp2-KO-Polk O/E-KCL22 cells treated with and without hydroxyurea. **F** Quantitative analysis of the percentage of γH2AX foci. A cell containing at least 10 foci was considered a foci-positive cell. **G** CML BC sample P#1217 and Ptbp2-KO-1217 cells were treated with and without hydroxyurea and probed with γH2AX antibody and DAPI. **H** Quantitative analysis of the percentage of γH2AX foci. A cell containing at least 10 foci was considered a foci-positive cell. **I** Percentage of cell viability in KCL22-NTC, Ptbp2-KO-KCL22, KU812-NTC, Ptbp2-KO-KU812, LAMA84, and Ptbp2 O/E LAMA84 cells treated with hydroxyurea at time points of 2 h, 4 h, 6 h, 8 h, and 12 h, respectively.

Polk, a TLS enzyme member, is known for bypassing DNA groove adducts. To investigate Ptbp2’s role in Polk-mediated DNA repair, KCL22-NTC and KU812-NTC, along with Ptbp2-KO-KCL22 and Ptbp2-KO-KU812 cells, were treated with 2 mM hydroxyurea (HU) for 4 h, followed by recovery of 12 h. HU is known to transiently stall replication by disturbing the dNTP pool. The comet assay showed increased comet tail length in Ptbp2-KO-KCL22 cells compared to KCL22-NTC cells after HU treatment, indicating greater DNA damage (Fig. 3A, middle panel compared to the upper panel). We observed a significant increase in the comet tail with HU treatment in the Ptbp2-KO-KU812 cells compared to the KU812-NTC cells and in the vector-LAMA84 cells compared to the Ptbp2-O/E-LAMA84 cells (Supplementary Fig. 3A, C). The bar diagram represents the change (Supplementary Fig. 3B, D). Overexpression of Polk in the Ptbp2-KO-KCL22 generated the Ptbp2-KO-Polk-O/E KCL22 cells (Fig. 3B), which were subjected to the comet assay after treatment with HU. They exhibited a small comet tail, almost identical to KCL22-NTC cells (Fig. 3A, lower panel). The bar diagram (Fig. 3C) represents the change. Thus, we demonstrate the significance of the Ptbp2-Polk axis in the DNA damage repair pathway.

Phosphorylation of the Ser-139 residue of the histone variant H2AX, forming γH2AX, is an early cellular response to the induction of DSBs [16]. The γH2AX foci were higher in the Ptbp2-KO-KCL22 compared to the KCL22-NTC cells when treated with HU (2 mM) (Fig. 3D, 4th panel compared to the 2nd panel). However, little change in the γH2AX foci was observed in the Ptbp2-KO-Polk-O/E KCL22 cells with or without HU treatment (Fig. 3D, 5th and 6th panels, respectively). The percentage of γH2AX foci is represented in the graph (Fig. 3F). The Ptbp2-KO-KCL22 cells, following the γH2AX foci, showed a higher expression of γH2AX in western blot as compared to the KCL22-NTC when treated with HU (Fig. 3E, middle panel, 4th lane); however, no change was observed when Polk was overexpressed in the KO cells (Fig. 3E, middle panel, 6th lane). The γH2AX foci were higher in vector-LAMA84 cells compared to the Ptbp2-O/E-LAMA84 cells when treated with or without HU (Supplementary Fig. 3E). The percentage of γH2AX foci and the protein expression are represented, respectively (Supplementary Fig. 3F, G). A similar observation was found in KU812-NTC and Ptbp2-KO-KU812 cells (Supplementary Fig. 3H). The percentage of γH2AX foci and the protein expression are also represented (Supplementary Fig. 3J, K). PBMC isolated from the CML BC sample (P#1217) were treated with or without HU for 4 h, followed by a recovery of 12 h. γH2AX foci were significantly higher in the Ptbp2 KO cells than in the NTC cells (Fig. 3G, last panel) upon HU treatment. The percentage of γH2AX foci is represented in the graph (Fig. 3H). We treated KCL22-NTC and KU812-NTC, as well as Ptbp2-KO-KCL22 and Ptbp2-KO-KU812 cells, along with vector-LAMA84 and Ptbp2-OE-LAMA84 cells, with HU for 2, 4, 6, 8, and 12 h. The cells were allowed to proliferate in fresh media for 6 days. 50% decrease in cell viability was observed in the Ptbp2-KO-KCL22, Ptbp2-KO-KU812, and the vector-LAMA84 cells (Fig. 3I). HU treatment, increased apoptosis in Ptbp2-KO- KCL22 and KU812 cells and in vector LAMA84 cells (Supplementary Fig. 4). These data suggest that the Ptbp2-Polk axis promotes DNA repair pathway and protects cells from apoptosis albeit at the cost of genomic instability, however, if attenuated, promotes apoptosis.

### PTBP2 regulates the MRE11-ATM-CHK2 axis via DNA polymerase kappa

**Fig. 4:**
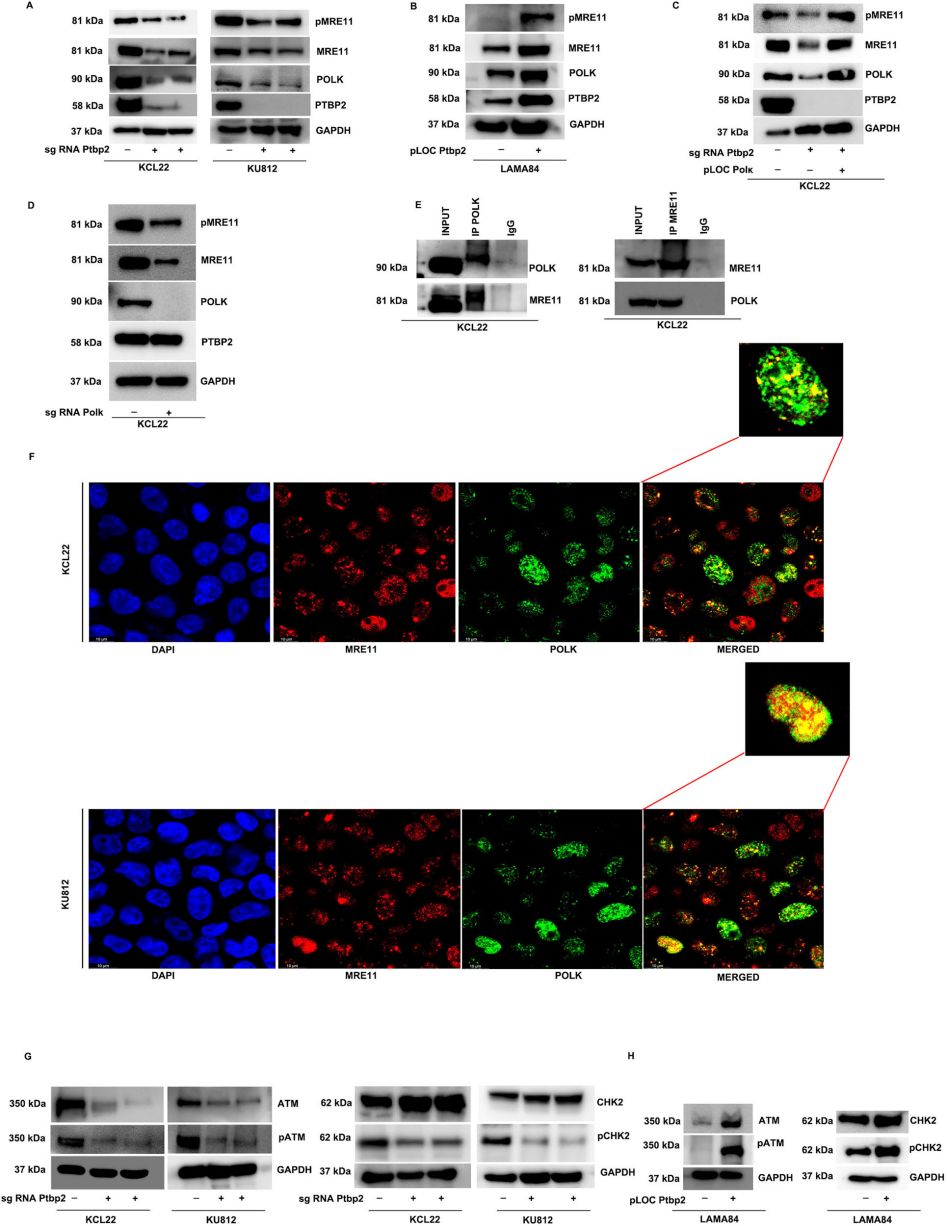
PTBP2 regulates the MRE11-ATM-CHK2 axis. **A** Mre11, pMre11, and Polk were expressed in WT NTC and two Ptbp2-KO-KCL22 and KU812 cell clones. **B** Expression of Mre11, pMre11, and Polk in LAMA84 and LAMA84 O/E Ptbp2 cells. **C** Mre11, pMre11, and Polk expression in WT NTC, Ptbp2-KO-KCL22, and Ptbp2-KO-Polκ-O/E-KCL22 cells. **D** Expression of Mre11, pMre11 in Polk ablated KCL22 cells. **E** Western blot analysis of co-immunoprecipitation of Polk and Mre11. **F** Representative images of cells co-immunofluorescence probed with anti-Mre11 (red) and anti-Polk (green) are shown in KCL22 and KU812 cells. **G** Protein expression of ATM, pATM, Chk2, and pChk2 in WT NTC and two different Ptbp2-KO-KCL22 and KU812 cell clones. **H** Protein expression of ATM, pATM, Chk2, and pChk2 in LAMA84 and Ptbp2-O/E-LAMA84 cells.

MRE11 serves as a primary sensor and recruits ATM, which in turn activates CHK2 via phosphorylation. MRE11 expression and phosphorylation were significantly reduced in Ptbp2-KO cells versus NTC in KCL22 and KU812 cells (Fig. 4A). Similarly, the overexpression of Ptbp2 in LAMA84 cells was positively correlated with the expression of MRE11 and pMRE11 (Fig. 4B). Co-immunoprecipitation showed no interaction between Ptbp2 and MRE11 (Supplementary Fig. 2B). The expression of MRE11 and pMRE11 was rescued when Polk was overexpressed in Ptbp2-ablated KCL22 cells (Fig. 4C). Ablation of Polk from the KCL22 cells decreased the expression of MRE11 and its phosphorylation thus confirming its regulation by Polk (Fig. 4D). To examine regulation of MRE11 by Polk, we immunoprecipitated Polk and assessed the co-immunoprecipitation (Co-IP) for MRE11, as well as conducted the reverse Co-IP. MRE11 co-immunoprecipitated with Polk and vice versa (Fig. 4E, left and right panels). This data was further validated as Polk and MRE11 were found to colocalize. The colocalization of Polk and MRE11 was confirmed in the CML cell lines KCL22 and KU812, as evidenced by Pearson correlation coefficients of 0.623 and 0.781, respectively. Similarly, Mander’s coefficients for KCL22 were 0.721 and 0.621, while for KU812 were 0.622 and 0.701, further supporting their interaction (Fig. 4F).

We investigated ATM-CHK2 expression and found that MRE11 recruits ATM to DNA damage sites. In KCL22 and KU812 cells, ATM-CHK2 phosphorylation was significantly lower in Ptbp2-KO cells compared to NTC cells (Fig. 4G). Ectopic Ptbp2 expression led to higher ATM-CHK2 phosphorylation in LAMA84 cells (Fig. 4H). Our results indicate that Ptbp2 stabilizes Polk’s 3′ UTR in CML-BC, boosting Polk-MRE11 interaction and error-prone DNA repair. Thus, we demonstrated that loss of Ptbp2 destabilizes Polk, turns off the ATM-CHK2 pathway, and triggers apoptosis.

### PTBP2 protects the stalled replication forks from degradation and promotes genomic instability and chromosomal aberrations

**Fig. 5:**
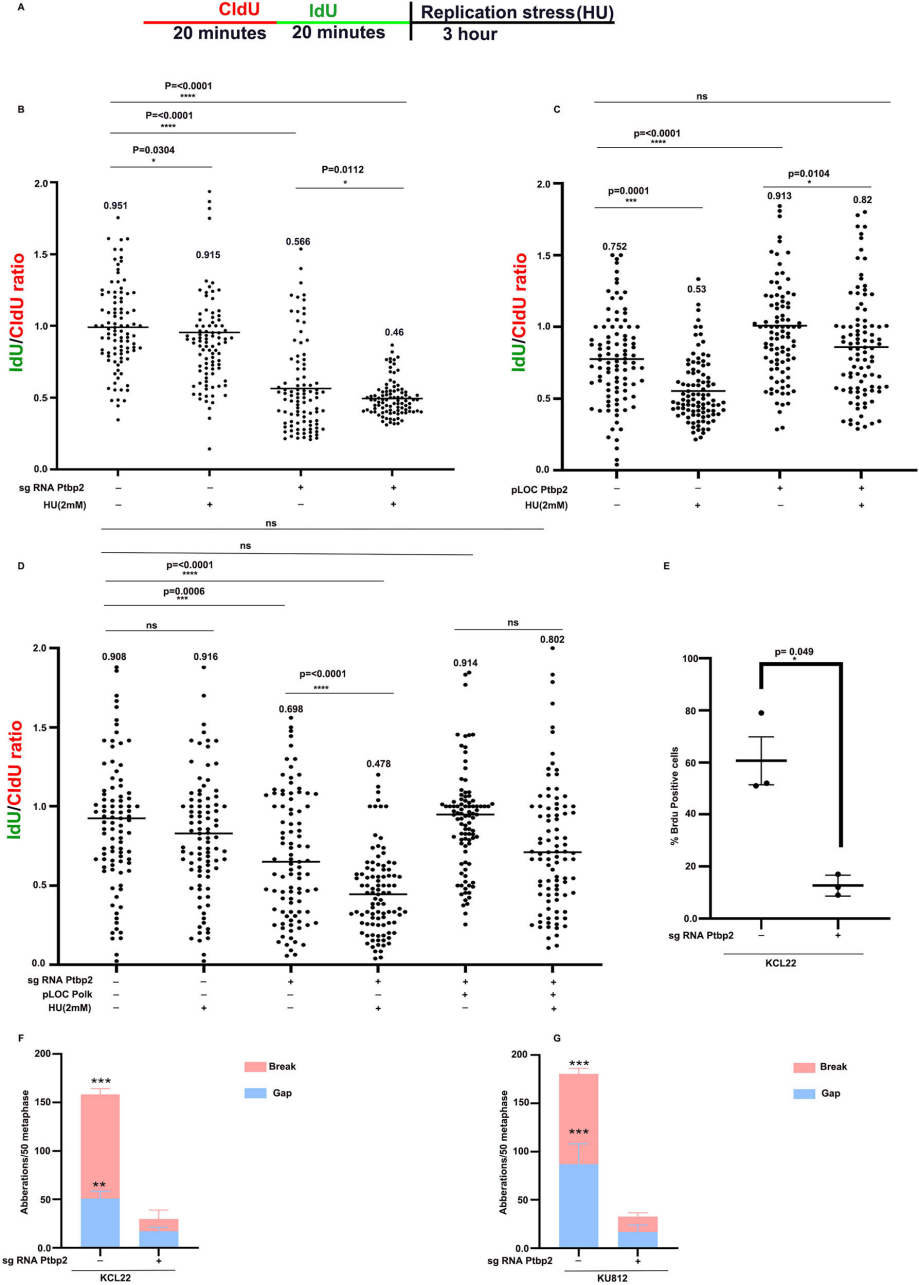
PTBP2 protects the stalled replication forks from degradation and promotes genomic instability. **A** Schematics for labeling cells with CIdU and IdU. **B** Ratios of IdU versus CIdU in the WT NTC, Ptbp2-KO-KCL22 cells treated with and without hydroxyurea. **C** Ratio of IdU versus CIdU in the LAMA84 and LAMA84 Ptbp2 O/E cells with or without hydroxyurea treatment. (NS not significant). One hundred replication forks were analyzed for each genotype. **D** Ratio of IdU versus CIdU in the KCL22-NTC, Ptbp2-KO-KCL22, and Ptbp2-KO-Polk-O/E-KCL22 cells treated with and without hydroxyurea. (NS, not significant). **E** Quantification of the percentage of Brdu-positive cells. **F**, **G** Aberrations were counted per 50 metaphases, specifying breaks and gaps in each of the following: KCL22-NTC, Ptbp2-KO-KCL22, KU812-NTC, and Ptbp2-KO-KU812.

To gain a better understanding of the Ptbp2’s role in DNA replication, a DNA fiber assay was used. Newly synthesized DNA was labeled with CIdU (red) before and ldU (green) after HU treatment. Retention of the IdU label after HU treatment measures the stability of stalled forks. KCL22 and the KO counterpart cells were labeled with CIdU (red) and IdU (green), and the active forks were stalled with HU (Fig. 5A). The depletion of Ptbp2 reduced the IdU/CIdU ratio to 35–45% in HU treated cells as compared to the NTC control cells (Fig. 5B). In Ptbp2 overexpressing LAMA84 cells concerning Vector-LAMA84 cells, replication forks were protected from degradation upon HU treatment, with an IdU/CIdU ratio close to 1 (Fig. 5C). Furthermore, the overexpression of Polk in Ptbp2 depleted cells reversed the effect caused by the Ptbp2 depletion as the IdU/CIdU ratio was found to be close to 1 indicating the replication forks were protected from fork degradation facilitating genomic instability (Fig. 5D).

We investigated the effect of Ptbp2 during the S phase by analyzing bromodeoxyuridine (BrdU) incorporation in asynchronous Ptbp2-ablated and NTC cells. BrdU incorporation was higher in NTC cells (Fig. 5E). We also examined the role of Ptbp2 in chromosomal aberrations using the sister chromatid exchange (SCE) assay in NTC and Ptbp2-ablated KCL22 and KU812 cells. Ptbp2 ablation showed a reduced number of breaks and gaps, demonstrating fewer chromosomal aberrations, relative to wild-type control cells in KCL22 and KU812 cells (Supplementary Fig. 5A, B). The graphical representation of breaks and gaps is represented in Fig. 5F, G. Collectively, the results suggest that Ptbp2 may contribute to genomic instability, disease progression, and the survival of malignant cells.

### MRE11 association at the replication forks depends on Polk-Ptbp2

**Fig. 6:**
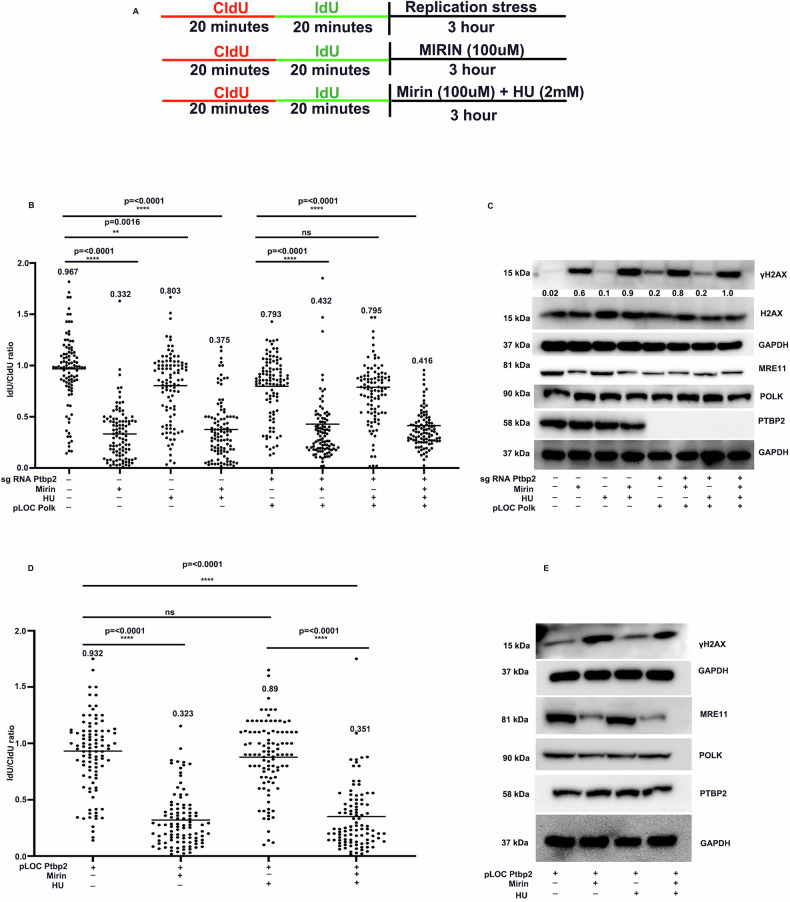
MRE11 association at the replication fork depends on POLK-PTBP2. **A** Schematic for labeling cells with CIdU and IdU, followed by treatment with hydroxyurea, mirin, and hydroxyurea + mirin, respectively. **B** Ratio of IdU versus CIdU in the KCL22-NTC, Ptbp2-KO-KCL22, and Ptbp2-KO-Polk O/E-KCL22 treated with hydroxyurea, mirin, and hydroxyurea + mirin, respectively. (NS not significant). One hundred replication forks were analyzed for each genotype. **C** Protein level expression of Ptbp2, Polk, Mre11, H2AX and γH2AX in the KCL22-NTC, Ptbp2-KO-KCL22, and Ptbp2-KO-Polκ-O/E-KCL22 treated with hydroxyurea, mirin, and hydroxyurea + mirin, respectively. Gapdh was used as a loading control. **D** Ratio of IdU versus CIdU in the LAMA84 and LAMA84 Ptbp2 O/E cells with hydroxyurea, mirin, and hydroxyurea + mirin, respectively. **E** Protein level expression of Ptbp2, Polk, Mre11, and γH2AX in the LAMA84 and LAMA84 Ptbp2 O/E cells with hydroxyurea, mirin, and hydroxyurea+mirin, respectively.

We found that Ptbp2 stabilizes replication forks through Polk, consistent with previous observations of Polk’s interaction with MRE11. We hypothesized that MRE11 stabilizes the stalled forks. To test this, we used the MRE11 inhibitor mirin and measured nucleotide incorporation in KCL22-NTC, KCL22+mirin, KCL22 + HU, and KCL22 + HU+mirin (Fig. 6A). We observed a significant decrease in IdU tract lengths in KCL22 cells treated with mirin and mirin+HU compared to the NTC and HU-treated groups. In Ptbp2-KO-Polk O/E KCL22 cells, Polk overexpression increased IdU tract length, treatment with mirin and mirin+HU reduced it (Fig. 6B). The replication fork arrest was associated with the expression of increased γH2AX in the protein level in the KCL22 cells treated with mirin, mirin+HU, Ptbp2-KO-Polk O/E KCL22 cells treated with mirin and mirin+HU however, KCL22-NTC, KCL22-NTC HU, Ptbp2-KO-Polk O/E KCL22 cells showed less expression of γH2AX thus confirming the replication fork stability (Fig. 6C). The ratio of γH2AX/H2AX is presented in Fig. 6C. We assessed LAMA84 cells and Ptbp2 O/E LAMA84 for nucleotide incorporation in the presence of HU and found a significant decrease in IdU tract lengths in the LAMA84 cells treated with mirin and mirin+HU compared to the Ptbp2 O/E LAMA84 and HU treated group (Fig. 6D). Additionally, γH2AX protein expression was higher in the Ptbp2 O/E LAMA84 cells treated with mirin, confirming replication fork stabilization via MRE11 (Fig. 6E). To further assess the recruitment of MRE11 at the replication fork, we conducted colocalization studies with PCNA, a known marker of replication sites, and observed colocalization, indicating the presence of MRE11 at the replication forks (Supplementary Fig. 7A). Collectively, the data suggest that the Ptbp2-POLK-MRE11 association functions in maintaining the replication fork stability and is a potential mechanism for the survival of the malignant cells.

### PTBP2 localizes at the site of the DNA damage upon HU treatment

**Fig. 7:**
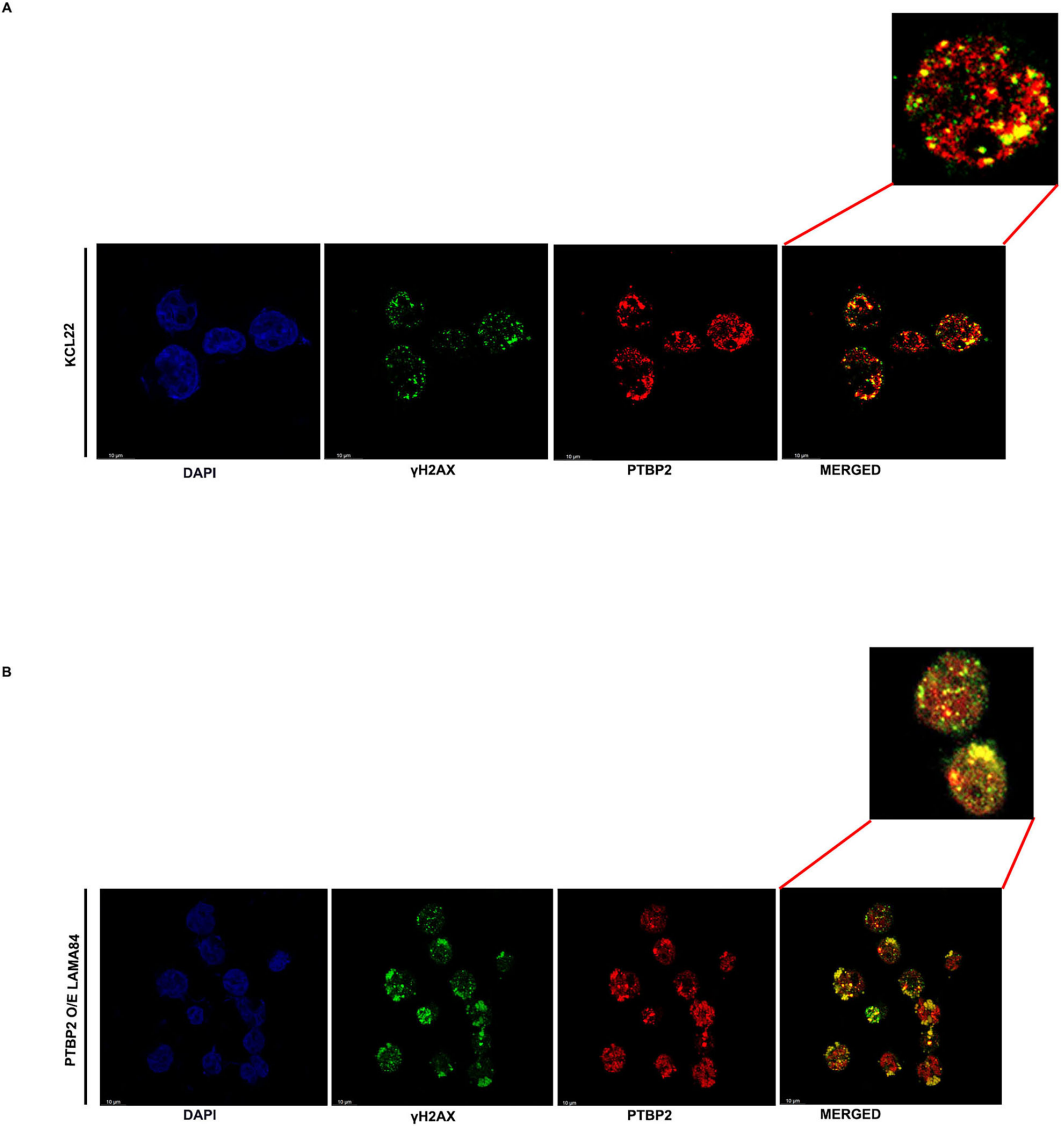
PTBP2 localizes at the site of DNA damage upon HU treatment. **A**, **B** Colocalization of Ptbp2 and γH2AX upon hydroxyurea treatment in KCL22 and LAMA84 Ptbp2 O/E cells.

We hypothesized that PTBP2 is recruited to stalled replication forks after HU-induced DNA damage. Consistently, 82% of KCL22-NTC cells showed PTBP2 colocalization with γH2AX, indicating replication fork stabilization (Fig. 7A). In Ptbp2 O/E LAMA84 cells, 72% showed PTBP2 and γH2AX colocalization after HU treatment, indicating replication fork stabilization in malignant cells (Fig. 7B).

### PTBP2 promotes tumor progression by increasing genomic instability

**Fig. 8:**
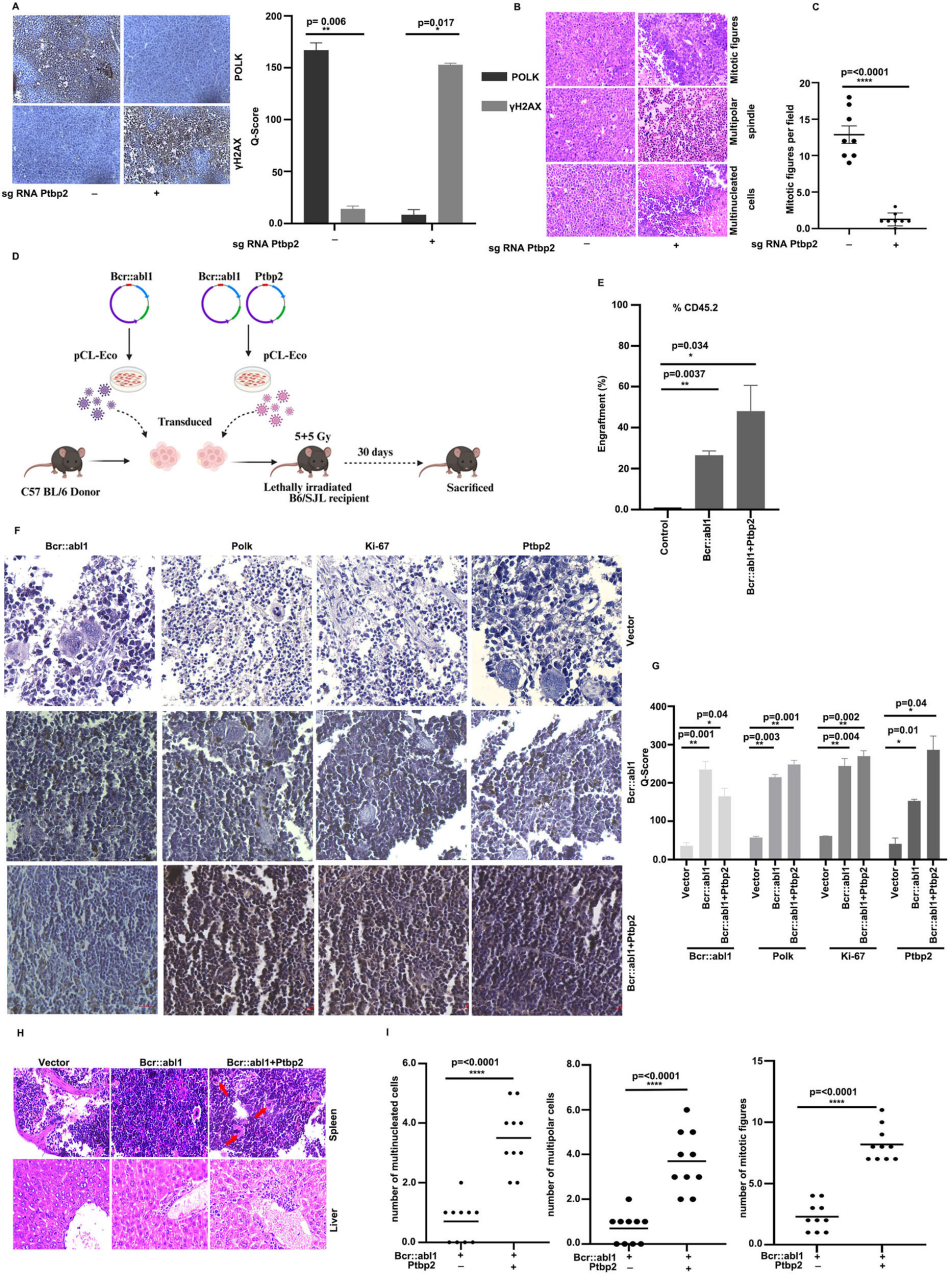
PTBP2 promotes tumor progression by increasing genomic instability. **A** Representative images of IHC of tumor tissues stained with Polk and γH2AX antibody and their respective Q-scores. **B** H&E staining of KCL22-NTC, Ptbp2-KO-KCL22 cell tumors. The first panel depicts mitotic figures; the second, a multipolar spindle; and the third, multinucleated cells. **C** Quantification of the mitotic figures per field. **D** Schematic illustration of Bcr::abl1 leukemia model development via bone marrow transplantation in mice. **E** Graphical representation of the percentage of engraftment of CD45.2 cells. **F** Representative images of IHC from the spleen of the respective groups probed with Polk, Ptbp2, Ki-67, and Bcr::abl1 antibodies. **G** Q-score of the respective groups. **H** H&E staining of the spleen and liver of the respective Bcr::abl1 and Bcr::abl1 + Ptbp2 transplanted mice groups. Red marks represent the multinucleated cells in each group. **I** Quantification of mitotic figures, multinucleated cells and multipolar spindles per field in Bcr::abl1 and Bcr::abl1+Ptbp2 transplanted mice.

Mice injected with KCL22-NTC cells developed large, aggressive tumors, whereas Ptbp2-KO-KCL22 cells produced smaller, less proliferative tumors with reduced Ki67 expression and less cellular pleomorphism [6]. Ptbp2 was markedly overexpressed in KCL22-NTC tumors compared to Ptbp2-KO-KCL22 tumors at both mRNA and protein levels, and Polk was also elevated in the tissue samples (Supplementary Fig. 8A, B). The expression of γH2AX was significantly higher in the Ptbp2-KO-KCL22 tumor than in the KCL22-NTC cells, thus indicating the unrepaired damage in the cells (Fig. 8A). The respective Q score is shown. Histopathological evaluation was done to assess the mitotic score and the number of typical and atypical mitotic figures in tumor tissues from KCL22 control and Ptbp2-KO-KCL22 group mice. The control group’s tumor samples showed numerous mitotic figures, indicating active karyokinesis or cell proliferation (Fig. 8B, upper panel). The number of mitotic figures per high-power field was approximately 10 times higher in tumors from the control group compared to the Ptbp2 knockout group, and is represented in the bar graph (Fig. 8C). The control group tumors had more abnormal mitotic figures, primarily tripolar or multipolar, with more than 2 chromosome clusters (Fig. 8B, middle panel). Asymmetrical mitotic figures with unequal-sized metaphase plates, considered atypical, were observed. Atypical mitotic figures indicate a disorganized arrangement of nuclear materials within dividing cells, reflecting genomic instability and associated with poor prognosis in various cancers. The control group also showed the presence of polyploid/multinucleated giant cells (Fig. 8B, lower panel), known to support tumor progression, therapy resistance, and cancer relapse.

We generated a BCR::ABL1 (CML) mouse model by transducing MSCV, BCR::ABL1, BCR::ABL1+Ptbp2 into C57BL/6J CD45.2 lineage-negative cells and transplanting them into 10 Gy-irradiated B6-SJL CD45.1 mice (Fig. 8D). After 11 days, the percentage of engraftment of CD45.2 was checked (Fig. 8E). Thirty days later the mice were sacrificed, and the spleen weight was found to be higher for the BCR::ABL1 and BCR::ABL1 + Ptbp2 cells concerning the vector control, however the spleen weight was not found to be statistically significant between BCR::ABL1 and BCR::ABL1 + Ptbp2 (Supplementary Fig. 8C). The expression of BCR::ABL1, Ptbp2, and Polk was found to be increased in the spleen of mice transplanted with BCR::ABL1+Ptbp2 (Supplementary Fig. 8D). Similarly, immunohistochemistry revealed the expression of Polk, Ptbp2 and Ki67 was higher in the BCR::ABL1+Ptbp2 group for both the spleen and the liver than in control and BCR::ABL1 mice groups (Fig. 8F). The Q score is represented respectively (Fig. 8G). Histopathological examination revealed normal histological architecture in the livers and spleens of the control group mice. The spleens of the BCR::ABL1 and BCR::ABL1 + Ptbp2 groups showed effacement of the typical architecture, characterized by increased infiltration of neoplastic myeloid cells, resulting in increased cellularity and a decrease in the red pulp proportion. The neoplastic cells were seen invading the splenic trabeculae and the capsule (Fig. 8H, upper panel). Cells with abnormal mitotic figures, multinucleated cells, and multipolar spindles were observed (Fig. 8I) and were significantly higher in the BCR::ABL1+Ptbp2 group, indicating increased genomic instability. The liver of these two groups showed infiltration of leukemic myeloid cells forming nodular collections in the sinusoids, especially in the portal area. The number and size of these nodules were comparatively higher in the BCR::ABL1+Ptbp2 group, indicating a more aggressive behavior of these cells. The surrounding hepatocytes exhibited degenerative and necrotic changes, which were more pronounced in the BCR::ABL1+Ptbp2 group (Fig. 8H, lower panel). Thus, observations from both mouse models suggest that the co-expression of Ptbp2 with BCR::ABL1 may represent one of the mechanisms that facilitate disease progression in CML by promoting genomic instability.

## Discussion

Cancer development is driven by the accumulation of mutations, resulting in increased genomic instability and the ability of cancer cells to evade cell death. In CML, the BCR::ABL1 kinase has been found to trigger DNA damage by promoting the production of reactive oxygen species, thereby causing genomic instability [7]. Additionally, data suggest that the accumulation of DNA damage, incorrect non-homologous end-joining (NHEJ) repair processes, and alterations in the DNA damage response play crucial roles in the progression to a more aggressive phase in CML patients [12]. This instability is associated with resistance to imatinib treatment and the accumulation of chromosomal abnormalities [17, 18]. Similarly, specific translocations in acute myeloid leukemia, such as FLT3/ITD, and myelodysplastic syndrome, such as Ras mutation, have been shown to induce DNA damage and disrupt the regulation of critical repair proteins [19]. Recently, we demonstrated that Ptbp2 is an oncogene in CML [6]. Presently, we demonstrate the importance of Ptbp2 in cancer progression, as its expression promotes Polk upregulation. Polk, an error-prone DNA polymerase, is upregulated in non-small cell lung carcinoma, melanoma, and glioblastoma, thereby stimulating DNA exchanges and promoting aneuploidy [13, 15, 20]. The overrepresentation of Polk contributes to genomic instability, thereby promoting tumorigenesis by enhancing cancer cell growth. Ablating Ptbp2 downregulates Polk, leading to increased H2AX phosphorylation and γH2AX foci, indicative of DNA damage, especially when treated with HU. HU is commonly used in CML to lower the WBC count before starting imatinib. HU initially resulted in stalled replication forks; however, after prolonged treatment, it collapses into DSBs [21]. POLK has been reported to play a critical role in fork restart during high-dose HU (2 mM) treatment [22]. Conversely, overexpressing Polk in Ptbp2 KO cells reduced H2AX phosphorylation and γH2AX formation upon HU treatment. Our findings suggest that the Ptbp2-Polk axis supports cell proliferation; Ptbp2 KO and HU treatment disrupts replicative potential and encourages apoptosis. The MRN complex is essential for detecting DNA damage and activating ATM at DNA foci [23, 24]. The MRE11 nuclease plays critical roles in responding to DNA DSBs at stalled, collapsed, or reversed replication forks [25–27]. Our findings revealed overexpression of MRE11 in NTC cells, and Ptbp2 stabilized the replication fork via Polk-MRE11; treatment of KCL22 cells with mirin was associated with an increased number of stalled replication forks. It has been reported that activation of the ATM-CHK2 cascade is essential for cell survival as it triggers a series of downstream pathways critical for DNA repair. ATM-CHK2 was upregulated in the presence of Ptbp2, whereas the genetic ablation of Ptbp2 resulted in the whole axis’s shutdown. Paull and Lee (2005) previously reported exciting findings on the physical interaction between MRE11 and ATM kinase [28]. We investigated the regulation of the MRN complex by Polk and found that it affects MRE11 but not RAD50 or NBS1. The interaction between Polk and MRE11 influences ATM and CHK2 expression, with Ptbp2 acting as a regulatory factor. Overexpression of Ptbp2-Polk leads to abnormal mitotic figures and giant cells, contributing to aggressive tumor development. This suggests that Ptbp2 promotes genomic instability via the POLK-MRE11-ATM-CHK2 axis, indicating a potential treatment avenue. Clinically, Polk is essential for managing cisplatin-induced DNA damage, as Polk-deficient tumors show delayed outgrowth and increased survival in treated mice [29]. Again, Polk mediates homologous recombination repair and temozolomide resistance in glioblastoma through the Rad17-dependent activation of ATR-CHK1 signaling, and deregulation of Polk sensitizes cells to temozolomide [15, 30]. Our findings highlight the problem and identify a potential therapeutic target: small-molecule-mediated depletion of Ptbp2 might reduce disease progression, as the transformation to the blastic phase was associated with Ptbp2 overexpression, offering hope for the development of effective CML blast crisis therapy.

## Materials and methods

### Cell lines and patient samples

The KCL22, K562, KU812, KYO-1, and LAMA84 CML cell lines, as well as the TF1, HEL, HNT34, and F36P AML cell lines, were cultured in RPMI medium 1640 supplemented with 10% FBS. The 32Dcl3 cells were cultured in RPMI 1640 medium supplemented with 10% FBS and IL-3. Meanwhile, the HEK293T human embryonic kidney cell line was cultured in Dulbecco’s modified Eagle medium supplemented with 10% FBS. All cell lines were mycoplasma-negative. Blood samples were collected from CML patients with ethical approval and written consent.

Further detailed information on experimental materials and methods is available in the Supplementary file.

## Supplementary information

Supplementary Figure 1

Supplementary Figure 2

Supplementary Figure 3

Supplementary Figure 4

Supplementary Figure 5

Supplementary Figure 6

Supplementary Figure 7

Supplementary Figure 8

Supplementary Fig. legends

Supplementary materials and methods

Supplementary table and legends

Raw unedited blots

## Data Availability

Research data supporting this publication are available on request.
