# Supplementary figures and images for: DNA polymerase kappa stabilized by Ptbp2 interacts with MRE11 and promotes genomic instability in leukemia
Source: Cell Death Discov. 2026 Feb 10;12:96. doi: 10.1038/s41420-026-02951-0 (PMC12920906; doi:10.1038/s41420-026-02951-0)

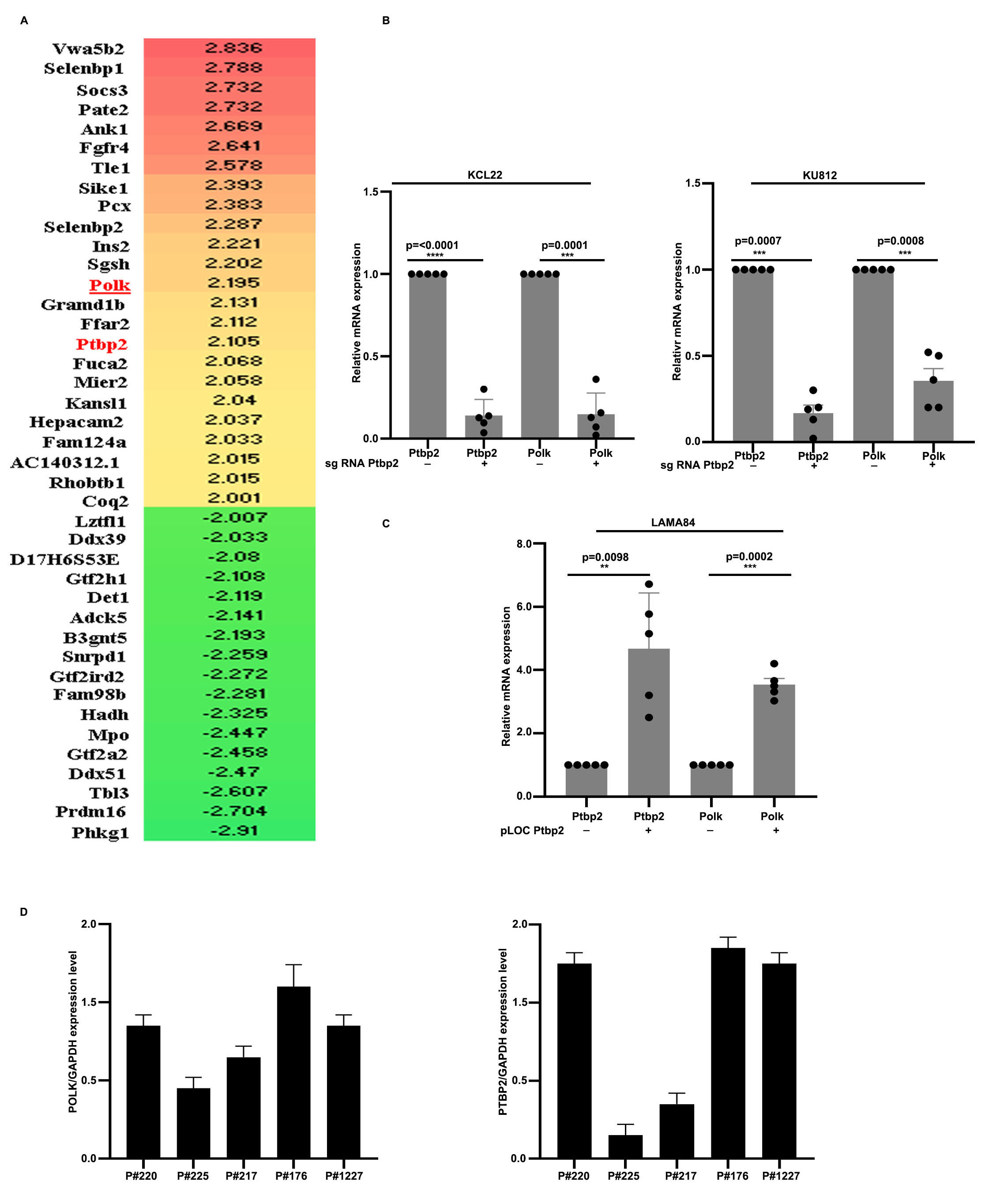

Supplement: Supplementary file 1 — Supplementary Figure 1 [file 41420_2026_2951_MOESM1_ESM.jpg]

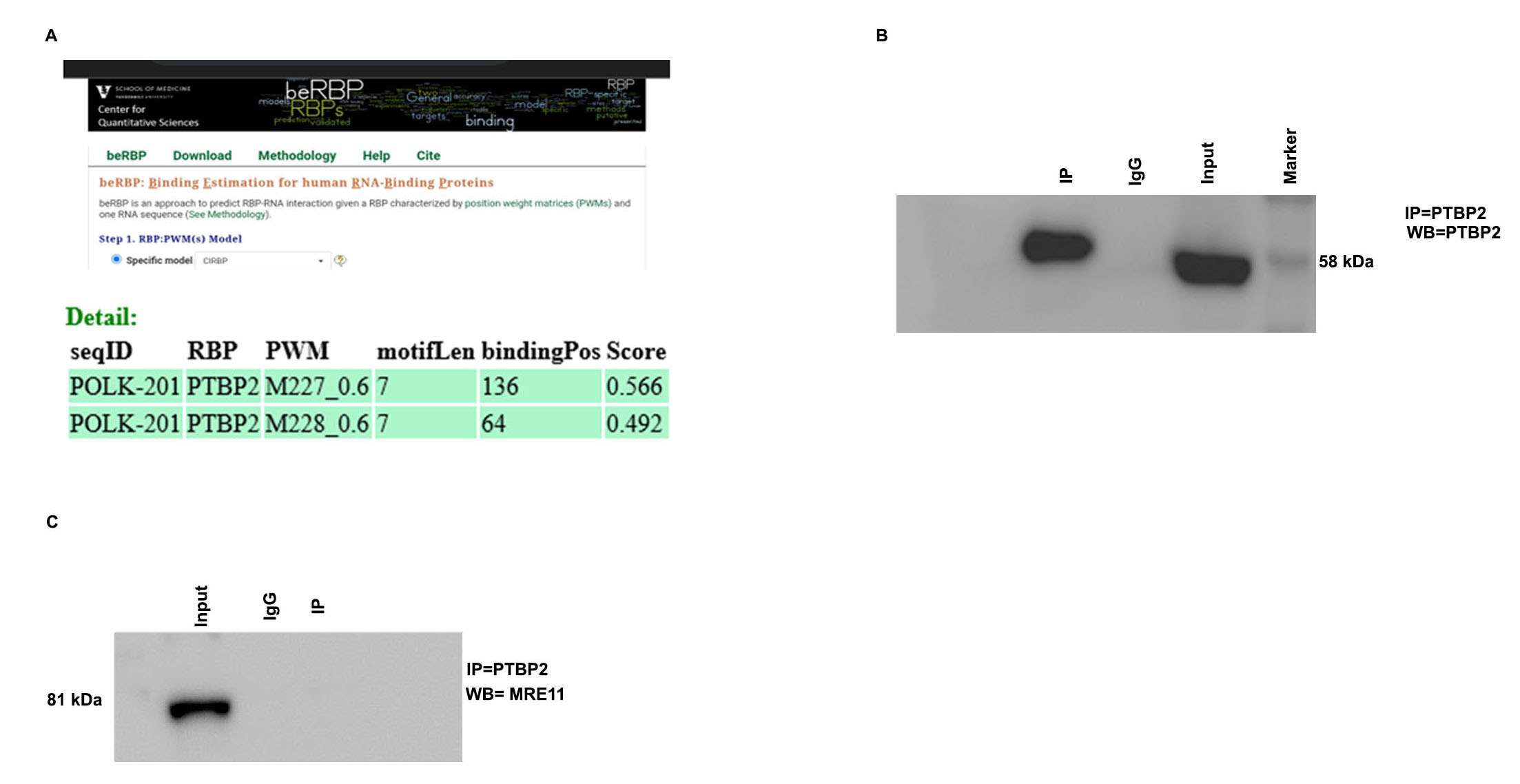

Supplement: Supplementary file 2 — Supplementary Figure 2 [file 41420_2026_2951_MOESM2_ESM.jpg]

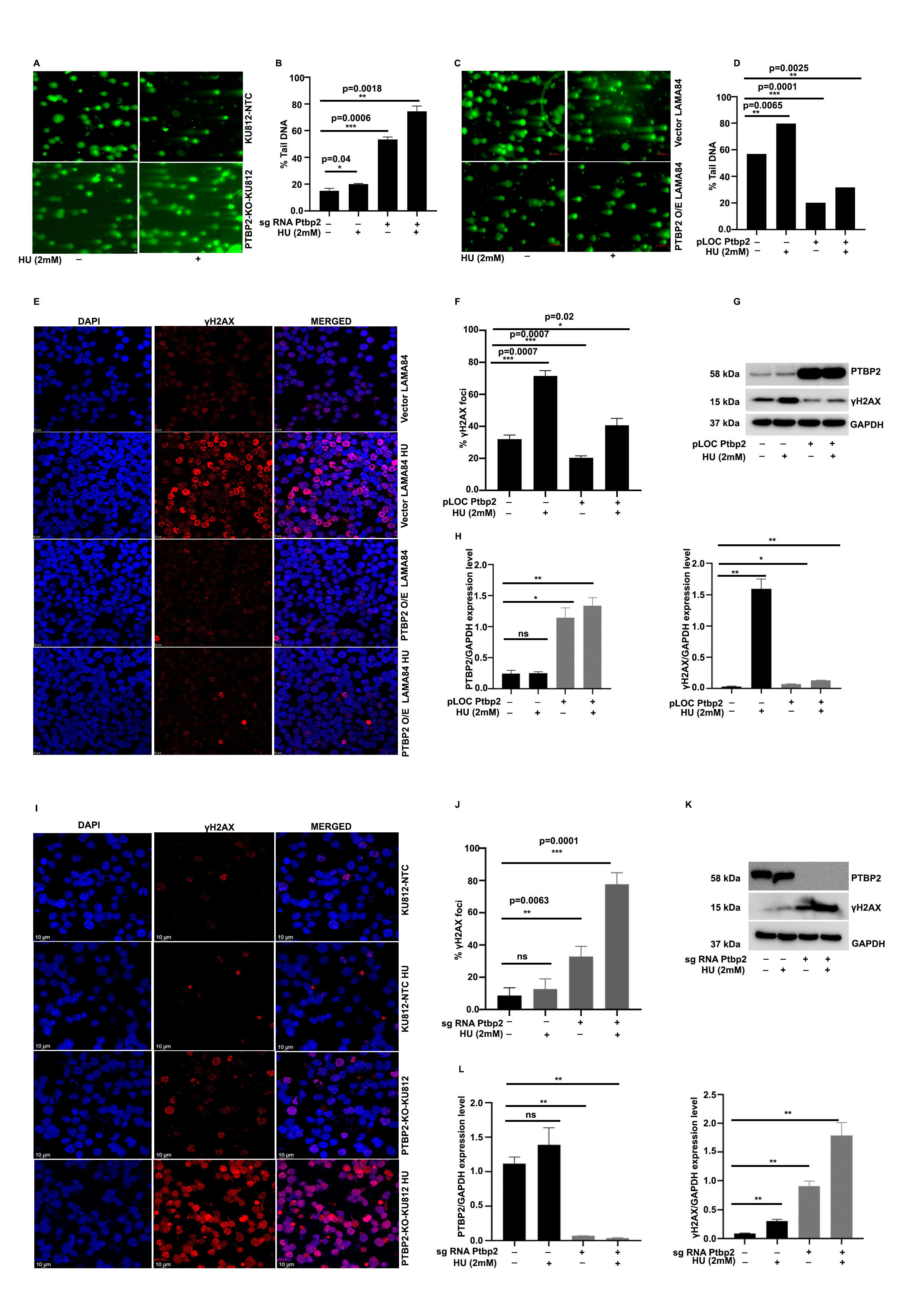

Supplement: Supplementary file 3 — Supplementary Figure 3 [file 41420_2026_2951_MOESM3_ESM.jpg]

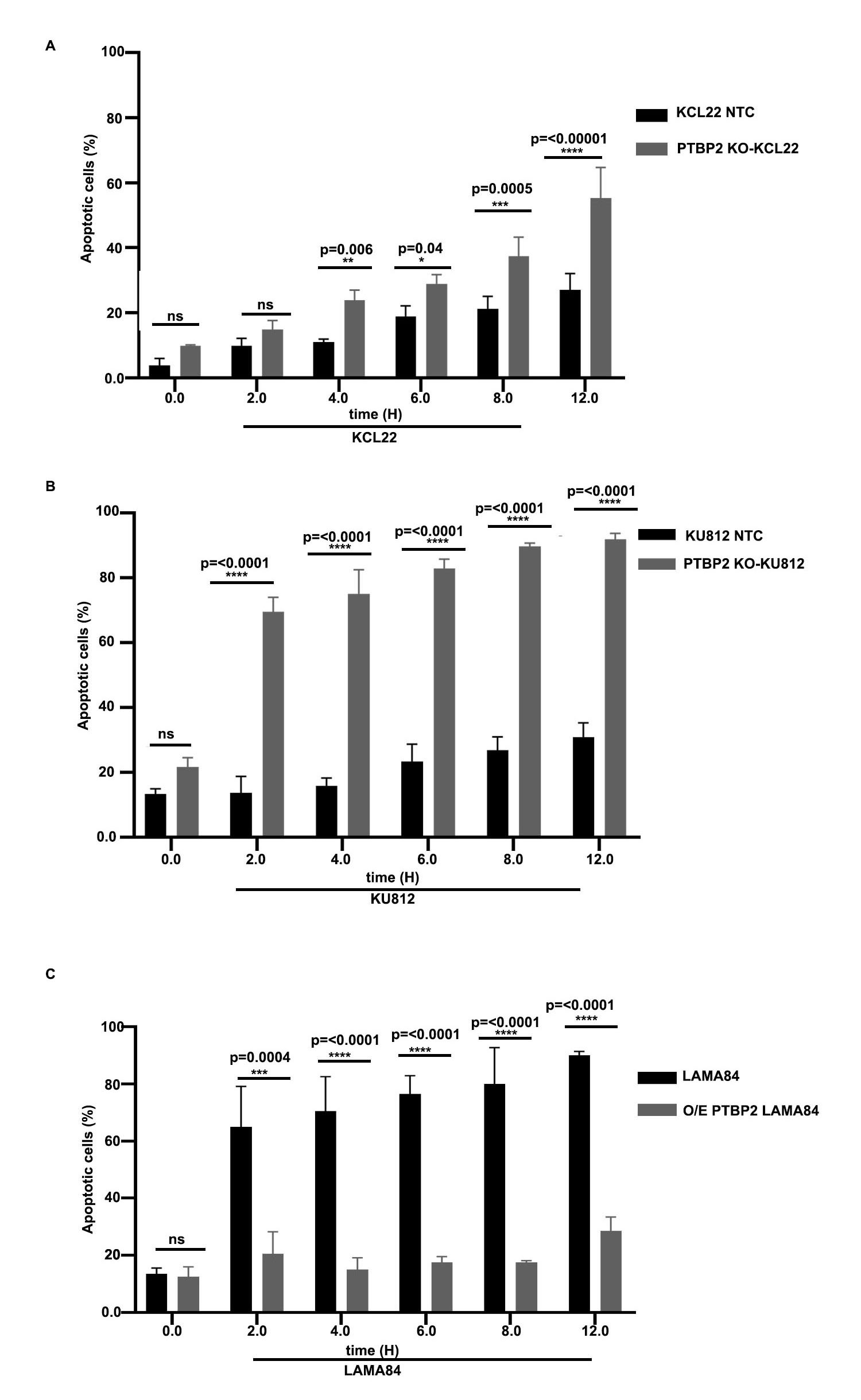

Supplement: Supplementary file 4 — Supplementary Figure 4 [file 41420_2026_2951_MOESM4_ESM.jpg]

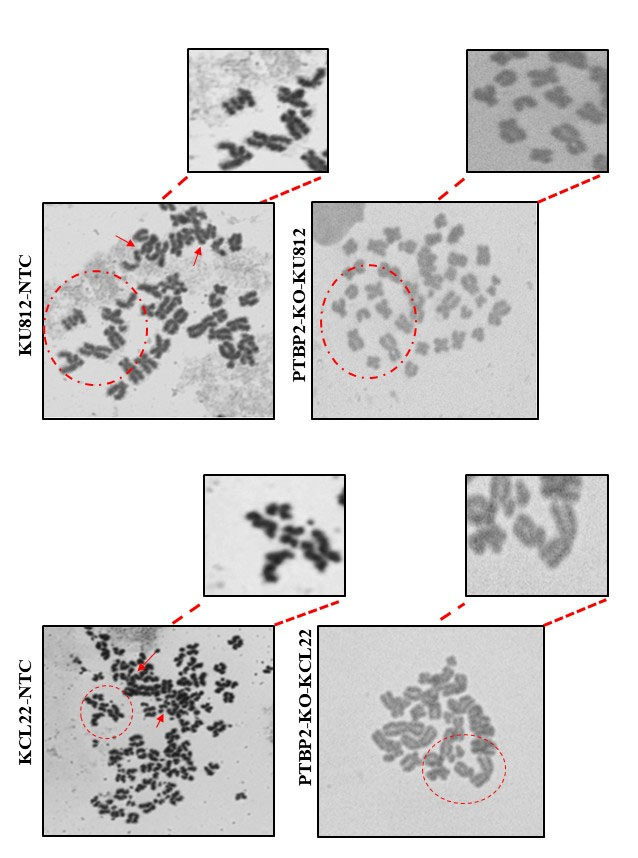

Supplement: Supplementary file 5 — Supplementary Figure 5 [file 41420_2026_2951_MOESM5_ESM.jpg]

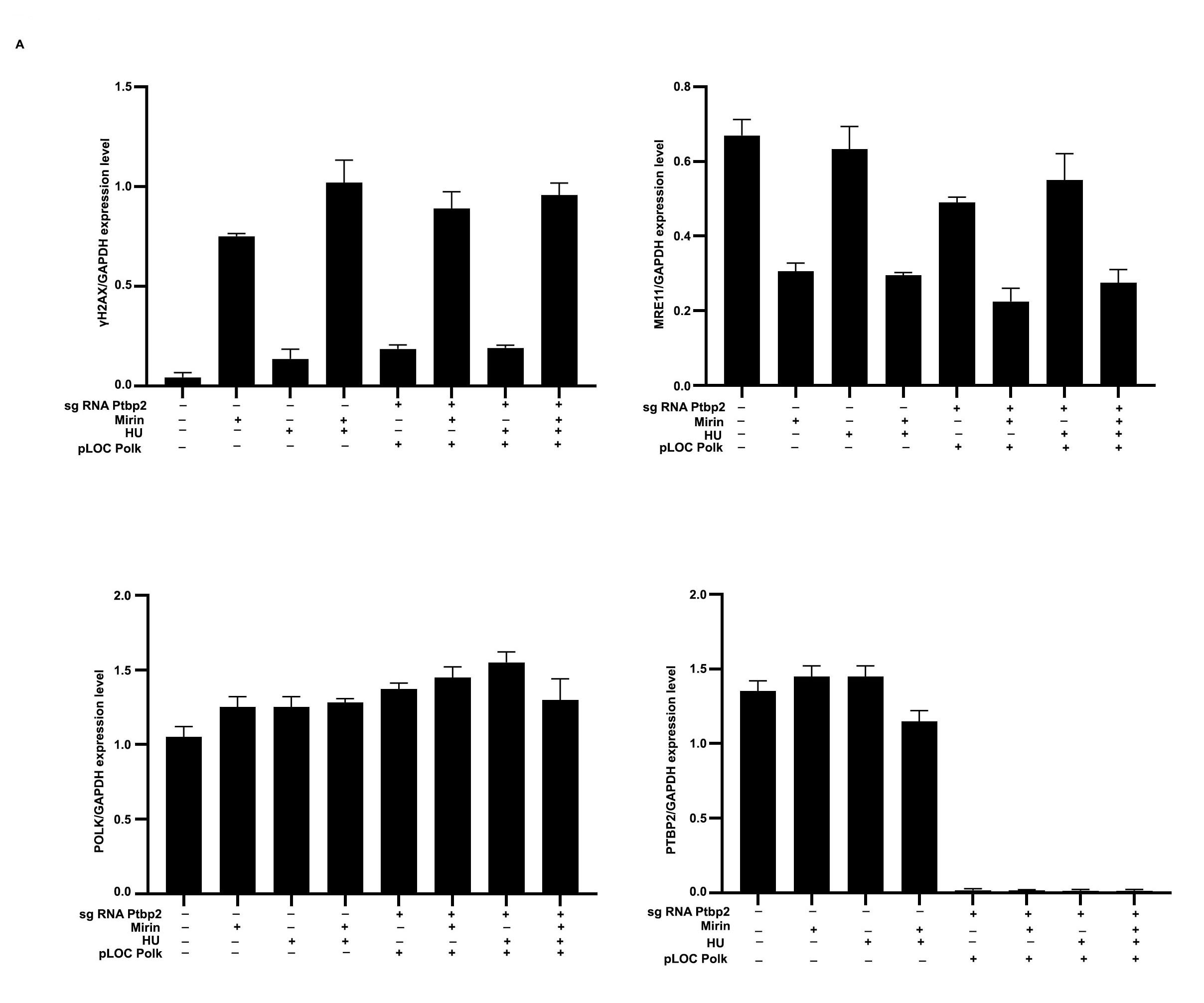

Supplement: Supplementary file 6 — Supplementary Figure 6 [file 41420_2026_2951_MOESM6_ESM.jpg]

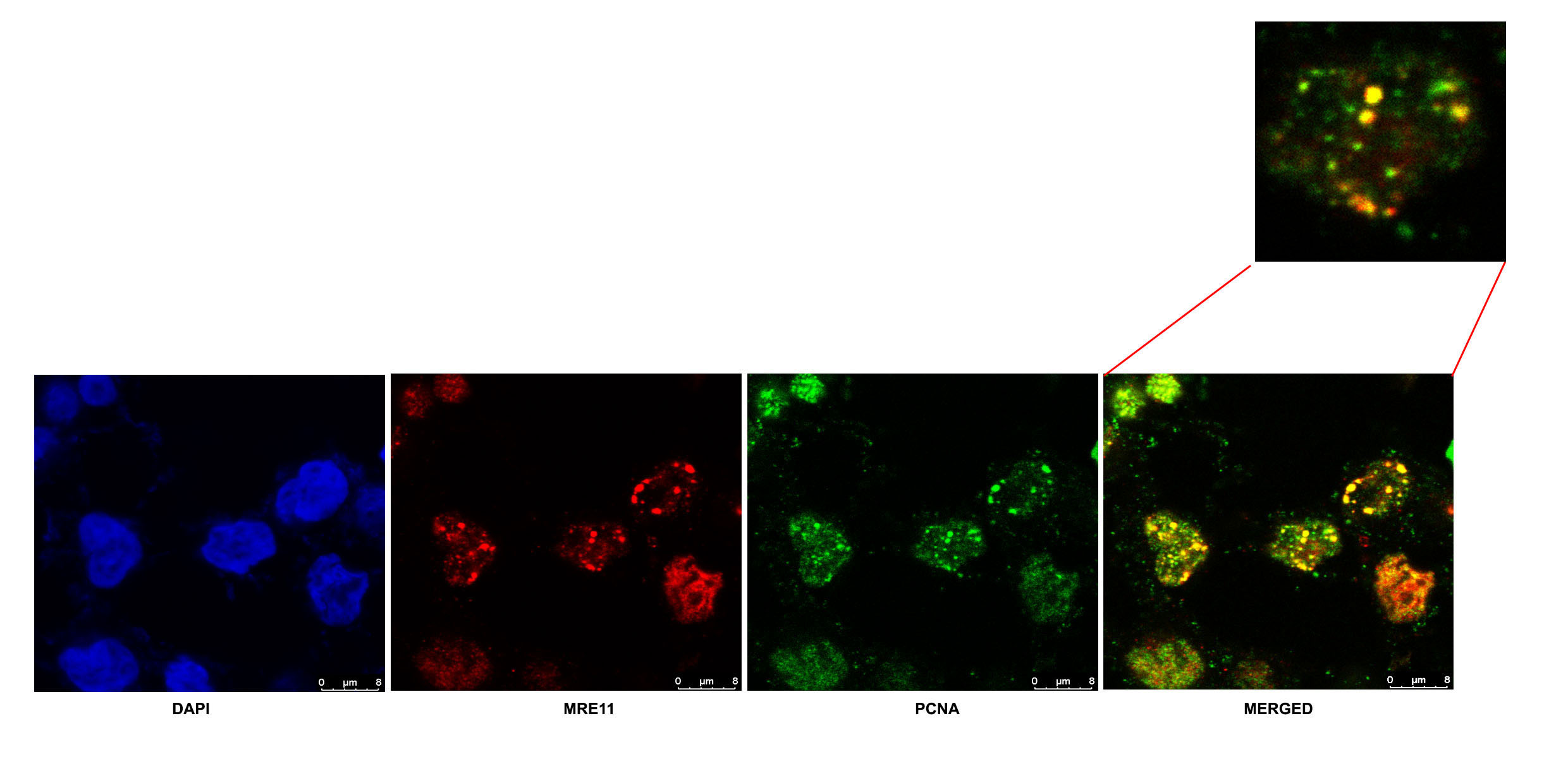

Supplement: Supplementary file 7 — Supplementary Figure 7 [file 41420_2026_2951_MOESM7_ESM.jpg]

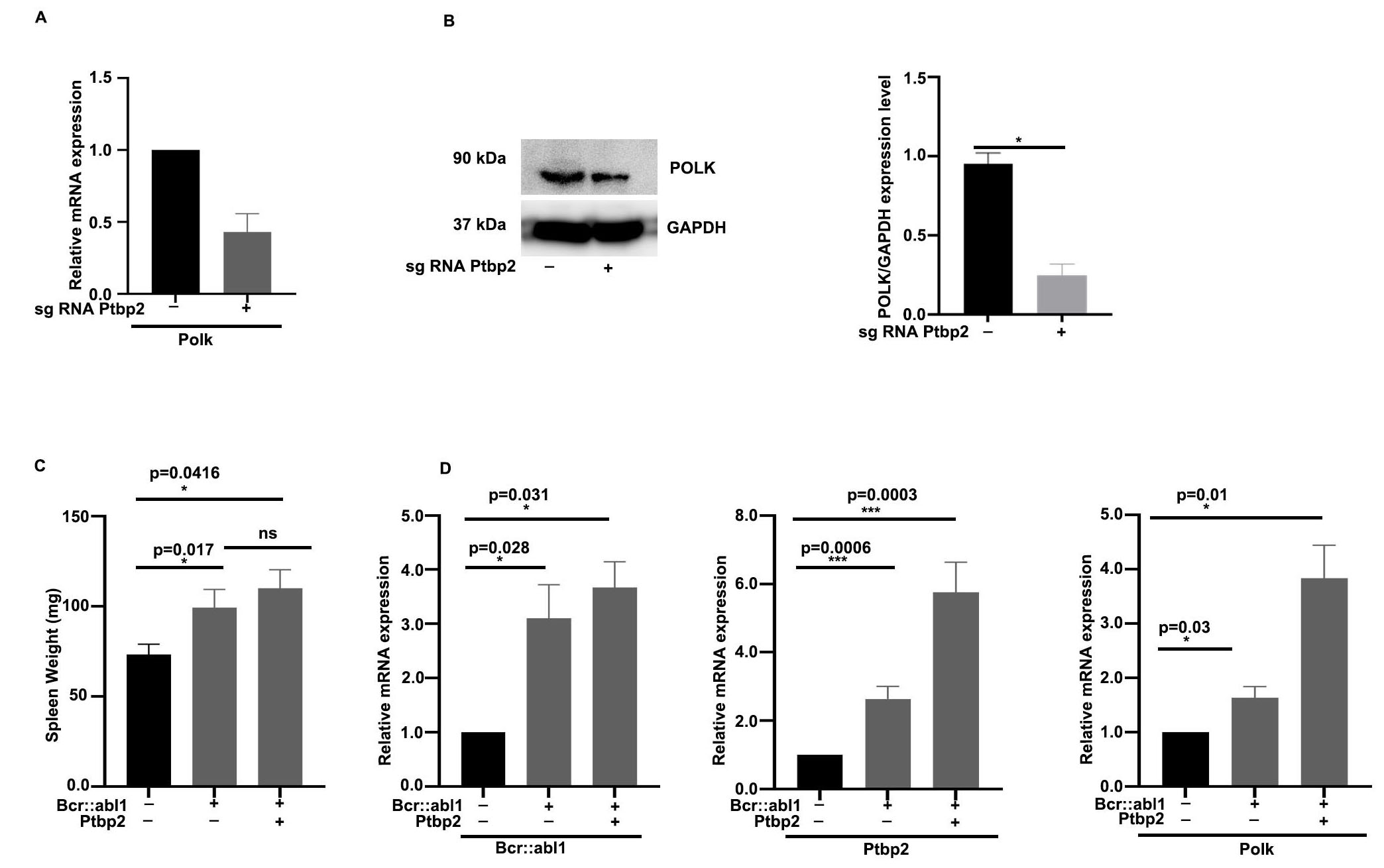

Supplement: Supplementary file 8 — Supplementary Figure 8 [file 41420_2026_2951_MOESM8_ESM.jpg]
