# Supplementary material for: DNA polymerase kappa stabilized by Ptbp2 interacts with MRE11 and promotes genomic instability in leukemia
Source: Cell Death Discov. 2026 Feb 10;12:96. doi: 10.1038/s41420-026-02951-0 (PMC12920906; doi:10.1038/s41420-026-02951-0)
Supplement: Supplementary file 9 — Supplementary Fig. legends [file 41420_2026_2951_MOESM9_ESM.docx]

**Supplementary Figure Legends**

Supplementary Figure 1

A) Gene expression analysis in vector 32Dcl3 and Ptbp2-32Dcl3 cells is represented in a heat map. B) mRNA expression of Ptbp2 and Polk in NTC and Ptbp2-KO KCL22 and KU812 cells. C) mRNA expression of Ptbp2 and Polk LAMA84 and LAMA84 Ptbp2 O/E cells. D) Densitometry analysis of Polk and Ptbp2 concerning Fig. 1G.

Supplementary Figure 2

A) Binding site prediction of Ptbp2 on Polk mRNA using beRBP. B) Validation of the immunoprecipitated Ptbp2 using Western blots. C) Expression of MRE11 following immunoprecipitation of Ptbp2.

Supplementary Figure 3

A) WT-NTC and Ptbp2-KO-KU812 cells were treated with or without hydroxyurea and subjected to alkaline comet assay. B) Quantification of the percentage of DNA tail. The data are presented as the mean ± SEM. C) LAMA84 and LAMA84 Ptbp2 O/E cells were treated with and without hydroxyurea and subjected to alkaline comet assay. D) Quantification of the percentage of DNA tail. The data are presented as the mean ± SEM. E) LAMA84 and LAMA84 Ptbp2 O/E cells treated with or without hydroxyurea and probed with γH2AX antibody and DAPI. F) Quantitative analysis of the percentage of γH2AX foci. A cell containing at least 10 foci was considered a foci-positive cell. G) Protein expression of γH2AX and Ptbp2 in LAMA84 and LAMA84 Ptbp2 O/E cells treated with and without hydroxyurea. H) Densitometry analysis of Ptbp2 and γH2AX, respectively. I) WT-NTC, Ptbp2-KO-KU812 cells treated with and without hydroxyurea and probed with γH2AX antibody and DAPI. J) Quantitative analysis of the percentage of γH2AX foci. A cell containing at least 10 foci was considered a foci-positive cell. K) Protein expression of γH2AX and Ptbp2 in WT-NTC, Ptbp2-KO-KU812 cells treated with and without hydroxyurea. L) Densitometry analysis of Ptbp2 and γH2AX, respectively.

Supplementary Figure 4

A) Graphical representation of the percentage of apoptotic cells in WT-NTC, Ptbp2-KO-KCL22 cells after treatment with hydroxyurea for 0, 2, 4, 6, 8, and 12h, respectively. B) Graphical representation of the percentage of apoptotic cells in WT-NTC, Ptbp2-KO-KU812 cells after treatment with hydroxyurea for 0, 2, 4, 6, 8, and 12h, respectively. Graphical representation of the percentage of apoptotic cells in LAMA84 and LAMA84 Ptbp2 O/E cells after treatment with hydroxyurea for 0, 2, 4, 6, 8, and 12h, respectively.

Supplementary Figure 5

A and B) Chromosomal aberrations in WT-NTC, Ptbp2-KO-KCL22, WT-NTC, and Ptbp2-KO-KU812 cells.

Supplementary Figure 6

A) Densitometry analysis of Ptbp2, γH2AX, Polk, and Mre11, respectively, concerning Fig. 6C.

Supplementary Figure 7

Representative images of cells co-immunofluorescence probed with anti-MRE11 (red) and anti-PCNA (green) are shown in KCL22 cells.

Supplementary Figure 8

A) mRNA expression of Polk in WT-NTC, Ptbp2-KO-KCL22 cell tumors. B) Western blot analysis of POLK from the protein isolated from KCL22-NTC, Ptbp2-KO-KCL22 cell tumors. GAPDH was used as a loading control. Densitometry analysis of Polk is represented. C) Comparison of spleen weights in mice transplanted with vector, Bcr::abl1, and Bcr::abl1+Ptbp2. D) mRNA expression of Bcr::abl1, Ptbp2, and Polk in Bcr::abl1 and Bcr::abl1+Ptbp2 transplanted mice.
