# Supplementary material for: DNA polymerase kappa stabilized by Ptbp2 interacts with MRE11 and promotes genomic instability in leukemia
Source: Cell Death Discov. 2026 Feb 10;12:96. doi: 10.1038/s41420-026-02951-0 (PMC12920906; doi:10.1038/s41420-026-02951-0)
Supplement: Supplementary file 10 — Supplementary materials and methods [file 41420_2026_2951_MOESM10_ESM.docx]

**Material and methods (contd.)**

**Generation of KO of Ptbp2 and Polκ in cell lines and patient samples and hydroxyurea treatment**

The study used lentivirus-based precision sgRNAs targeting Ptbp2 (6) and Polκ (Horizon, Cat. # VSGH12606-256402768) to knock out Polκ in KCL22 and KU812 cells, using puromycin as a selection marker. Peripheral blood mononuclear cells (PBMC) were isolated using the Ficoll gradient method. Knockout of *Ptbp2* was produced in the mononuclear cells by transducing specific sgRNA for *Ptbp2* exon 1 (**6**). RT-qPCR and Western blotting confirmed *Ptbp2* knockout. Cells were treated with hydroxyurea (HiMedia Cat#H0310) (2mM) for 4h, followed by a recovery of 12h.

**Generation of stable cell lines overexpressing PTBP2 and POLK**

Codon-optimized Ptbp2 cloned in the MSCV-Neo vector was co-transfected with the pCL-Eco vector in the 293T cells to generate the vector control and Ptbp2 retroviral particles. Subsequently, the virus was introduced individually into the murine myeloblastic progenitor cell line 32Dcl3, and clones were selected with neomycin. The study used lentivirus-based precision lentiORF PTBP2 w/stop codon to overexpress Ptbp2 (Horizon, Cat. #OHS5899-202618163) in LAMA84 cells with blasticidin as a selection marker (**6**). The coding region of *Polκ* was cloned into a lentivirus vector, and the lentivirus was produced by transfecting the Polκ plasmid along with the packaging plasmid pPAX2 and the envelope plasmid pMD2G into HEK293T cells using a standard transfection protocol. Furthermore, Ptbp2 KO cells were transduced with Polκ lentivirus in the presence of polybrene (8 µg/ml) and examined for GFP expression. GFP-positive cells were sorted, and POLK expression was checked using Western blot analysis.

**RNA isolation, microarray, cDNA preparation, and RT-qPCR**

RNA isolation was performed using TRIzol (Ambion, Cat#15596018) according to the manufacturer’s protocol. The quality of the RNA was checked in the Tape Station and subjected to microarray analysis using the Affymetrix GeneChip Mouse Exon 1.0 ST Array (Catalogue No: 900818) as per the manufacturer’s instructions. RT-qPCR was performed using the GoTaq qPCR Master Mix (Promega, Cat # A6002). GAPDH was used as a loading control. The oligos used for qRT-PCR in this study are listed in **Supplementary Table 1**.

**Western Blotting**

Isolation of cell protein lysate and Western blotting, as mentioned earlier (6), was done. The membrane was probed with the following antibodies: GAPDH (CST Cat# 5174S RRID: AB_2737054), POLK (Santa Cruz Cat# 166667 RRID: AB_2047559), PTBP2 (CST Cat# 15719S RRID: AB_1930420), MRE11 (CST Cat 4895S RRID: AB_398888), pMRE11 Ser 676 (CST Cat#4859 RRID: AB_2145096 ), CHK2 (CST Cat# 2662S RRID: AB_390016), pCHK2 Thr68 (CST Cat#2661 RRID: AB_1968482), γH2AX Ser 139 (CST Cat#2577 RRID: AB_420030). The following secondary conjugated antibodies were used: Anti-rabbit HRP-linked antibody (CST Cat#7074 RRID: AB_2099234), Anti-mouse HRP-linked antibody (CST Cat#7076 RRID: AB_331144), and H2AX (CST Cat#7076 RRID: AB_2115094)

**Immunofluorescence**

Cells cultured in RPMI with 10% heat-inactivated serum were attached to the coverslips coated with poly-l-lysine (Sigma, Cat# P4707). The coverslips with attached cells were fixed with a 1:1 methanol: acetone mixture for 20 min at -20°C. The coverslips were washed with 1x PBS, and blocking was performed with 3% BSA (Thermo Fisher Scientific) for 1 hour. The following antibodies were used: POLK (Santa Cruz, Cat#166667 RRID: AB_2047559), PCNA (CST, Cat 13110 RRID: AB_11218307), MRE11(CST, Cat 4895 RRID: AB_11218307), γH2AX Ser 139(CST, Cat# 80312 RRID: AB_3667015), γH2AX Ser 139 (CST, Cat#2577 RRID: AB_420030), PCNA (CST, #13110 RRID: AB_11218307), MRE11 (CST, #4895 RRID: AB_11218307) and γH2AX Ser 139 (CST, #2577 RRID: AB_420030). The coverslips were incubated overnight in a moist chamber at 4°C. Secondary antibodies Anti-rabbit 594 Alexa Fluor (Invitrogen, Cat#A11072 RRID: AB_3251387) and anti-mouse 488 Alexa Fluor (Invitrogen, Cat# A21202 RRID: AB_2341099) were used and incubated for 1h in the dark. The coverslips were stained with DAPI (Merck, Cat#10236276001) for 90 seconds and then mounted in the slides using mounting media (Invitrogen, Cat #S36938). The slides were visualized using a confocal microscope at 63X (Leica, Germany).

**Alkaline comet assay**

Cells were harvested, treated with Hydroxyurea (2mM), and blended with 0.5% low-melting agarose. The cells were then added to slides precoated with 1% agarose. The slides were lysed and denatured, then subjected to electrophoresis. The slides were stained with DAPI and viewed in a fluorescence microscope. The DNA tail percentage was measured using CASP software.

**Actinomycin D chase assay**

2.8x10^6^ KCL22, Ptbp2 KO KCL22, LAMA84, Ptbp2 O/E LAMA84 cells were maintained in RPMI containing 10%FBS in 5% CO_2_ incubator at 37 ℃. Cells were treated with Actinomycin D (Sigma, Cat#A4262) at 5 µg/mL for the following time points: 1h, 2h, 4h, 6h, 8h, and 12h. RNA isolation and cDNA preparation were performed according to the standard protocol. RT-qPCR was done using GAPDH as a control. The mRNA stability was calculated using GraphPad Prism 7.

**RNA immunoprecipitation (RIP)**

RNA immunoprecipitation was performed as described previously (**6**). RT-qPCR was used to quantify transcript levels.

**Luciferase reporter assay**

The 3’UTR of Polk, comprising the PTBP2 binding site, was cloned into a luciferase vector, and a luciferase reporter assay was performed as described (**6**). The binding site was mutated using site-directed mutagenesis.

### **Cell survival assay**

Cells (5000/well) were seeded in a 24-well plate. After treatment with hydroxyurea at 2 mM for different time points, cells were allowed to grow for 7 days. Cell survival was monitored using the MTT (Sigma, #M5655, 0.1 mg/mL) assay, with readings taken at 570 nm in a VICTOR Nivo^TM^ multimode reader (PerkinElmer, USA). Percent survival was calculated as NTC cells/ Ptbp2 KO cells*100.

**BrdU incorporation assay**

100 µM bromodeoxyuridine (Sigma, Cat#B5002) was incorporated into DNA for 20 min to visualize replication foci. Cells were fixed with 70% cold ethanol. Cells were denatured in 1.5 M HCl for 30 min and then incubated in 5% BSA, 0.5% Tween 20, and 5% FBS for 20 min. Primary Anti-Brdu (Merck, Cat#B2531 RRID: AB_10659710) antibody was added to the slides, which were precoated with poly-l-lysine (Sigma, cat# P4707), and incubated for 1h. Slides were washed with PBS-Tween20 and incubated with a secondary antibody Alexa Fluor 488 (Invitrogen, Cat#A21202 RRID: AB_2341099) for 1h.

**Metaphase spreads**

1x10^6^ cells were seeded in RPMI+10%FBS in a humidified chamber at 5% CO_2_ chamber at 37°C. Cells were treated with colchicine (Sigma, Cat#C9754) for 45 min before harvesting. Cells were treated with 0.056 M KCl for 30 min and then fixed with methanol: glacial acetic acid (3:1). The cell suspension was taken in a Pasteur pipette, and a single drop was released from a height onto the slide. The slides were allowed to air dry. The slides were stained with Giemsa (Himedia, Cat#TCL083) for 30 min. The images were acquired in an Apotome microscope (Zeiss).

**DNA Fiber assay**

The cells were labeled with 100 μM of CIdU (Sigma, Cat# C6891) and 100 μM of IdU (Sigma, Cat# I712) for the indicated times. HU was added, as shown in the result. Cells were harvested, lysed, and spread on superfrost slides. Cells were denatured with 2.5 M HCl and blocked with a blocking solution. The slides were incubated with primary antibodies, Rat anti-CldU 1:100 (Abcam, Cat#ab6326 RRID: AB_609568) and Mouse Anti-IdU 1:100 (Becton Dickinson, Cat# 347580 RRID: AB_2611275) for 2.5 hours at RT, washed with PBS, and incubated with Rat anti-Cy3 (Jackson Immuno Research, Cat#712-166-153 RRID: AB_608718), Mouse Alexa 488 (Molecular Probes, Cat#A11001 RRID: AB_1101761) secondary antibodies. Following mounting, slides were imaged using a confocal microscope at 63X (Leica, Germany).

**Animal study**

All animal protocols were performed with the approval of the Institute of Life Sciences ethics committee (ILS/IAEC-03-AH/05-14 and ILS/IAEC-159-AH/AUG-19). The subcutaneous tumor model, as described by Barik et al. (2024) (6), and the transplantation model using C57BL/6J CD45.2 and B6-SJL CD45.1 mice were employed according to the standard protocol. All mice were bred in the animal house facility. 8-10 weeks after inclusion, mice were included in the study; lineage-negative cells were isolated from C57BL/6J CD45.2 mice using a lineage-negative cell isolation kit (Miltenyi Biotech, Cat#130-110-470) and transduced with the MIGR1 vector, BCR::ABL, and BCR::ABL+Ptbp2 virus. Transduced bone marrow cells were transplanted into 8-10-week-old lethally irradiated (10 Gy) B6-SJL CD45.1 mice. The mice were randomly distributed in each group. Control mice were transplanted with lineage-negative cells transduced with the MIGR1 vector. After 30 days, the mice were sacrificed, and their livers, kidneys, and spleens were isolated and stored in formalin.

**Immunohistochemistry (IHC)**

IHC staining of the tissue was performed according to the protocol described previously (**6)**. Each slide marked one of the sections as a negative control, in which the primary antibody was omitted. Images were obtained using a Leica DM500 microscope (Leica, Germany). Antibodies used for the assay: Polk (Santa Cruz, Cat# 166667 RRID: AB_2047559), PTBP2 (CST, #Cat 15719 RRID: AB_1930420), γH2AX Ser 139 (CST, #2577 RRID: AB_420030), BCR::ABL junction (CST, #3908 RRID: AB_490962), Ki-67 (CST, #12202 RRID: AB_2722785).

**H&E staining**

Formalin-fixed and paraffin-embedded 4-5μm-thick sections were taken. The sections mounted on slides were washed three times with Xylene (Himedia, Cat# AS080), followed by rehydration in 100% ethanol, 95% ethanol, 80% ethanol, and 70% ethanol, each for 5 min. The slides were stained with Hematoxylin (Himedia, Cat# S058) for 2 minutes and then washed in tap water. The slides were stained with Eosin (Himedia, Cat#3007), dehydrated with 90% and 100% ethanol for 30 seconds each, and then with xylene for 30 seconds. The slides were mounted in DPX (Merck, Cat#06522) and imaged.

### **Statistical analysis**

GraphPad Prism 7.0 was used to analyze the data and create the graphs. Statistical analysis was performed using two-way ANOVA for grouped analysis or a paired *t*-test for 2 groups. All data are represented as the mean ± SEM. *, p < 0.05, * *, p < 0.01 and * **, p < 0.001 were considered as statistically significant. All experiments were performed at least three times with three biological repeats.
