# Supplementary material for: DNA polymerase kappa stabilized by Ptbp2 interacts with MRE11 and promotes genomic instability in leukemia
Source: Cell Death Discov. 2026 Feb 10;12:96. doi: 10.1038/s41420-026-02951-0 (PMC12920906; doi:10.1038/s41420-026-02951-0)

Fig.1

Uncropped & unedited western blots

1A

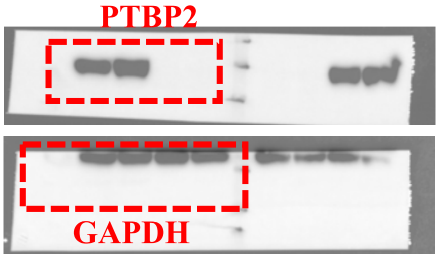

1C

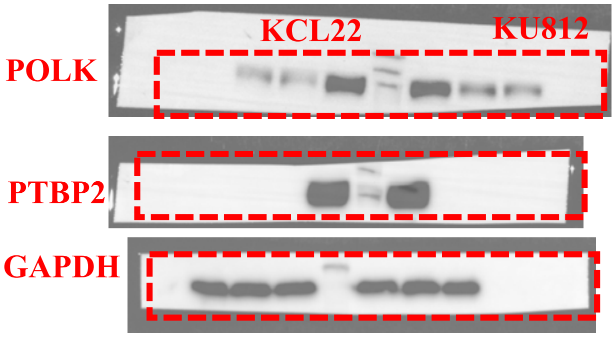

1D

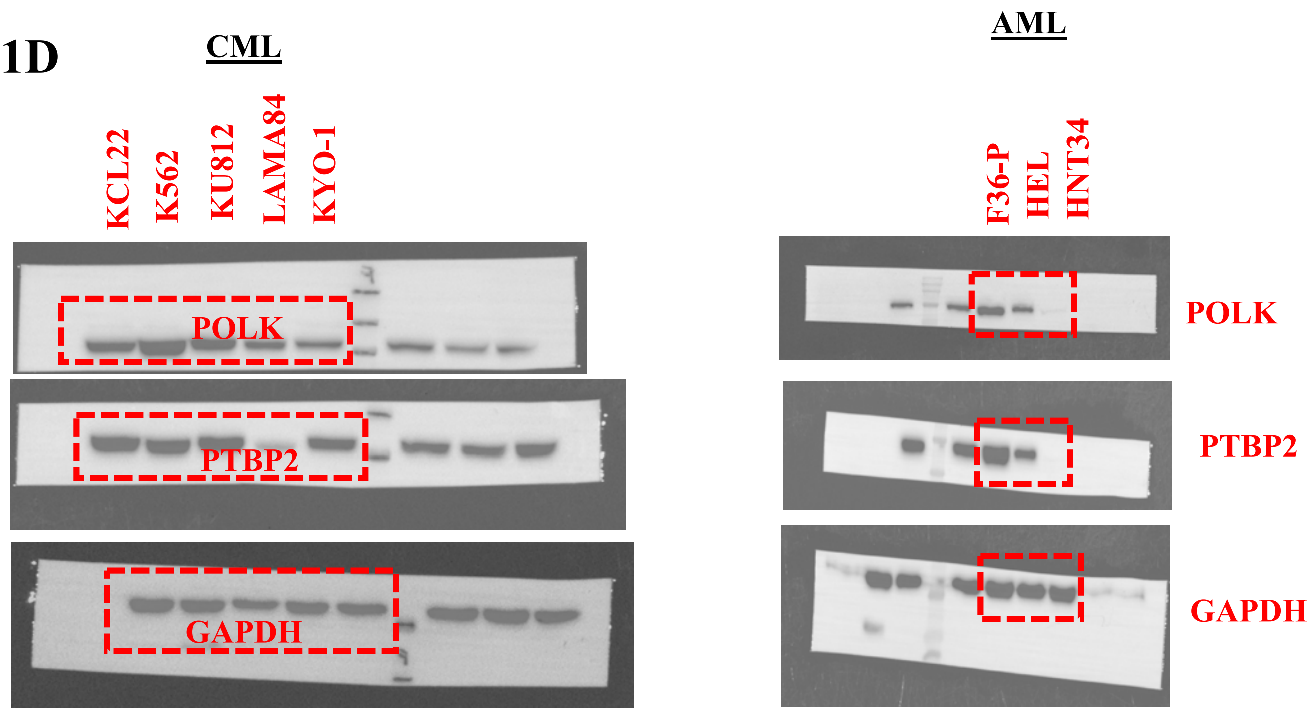

1E

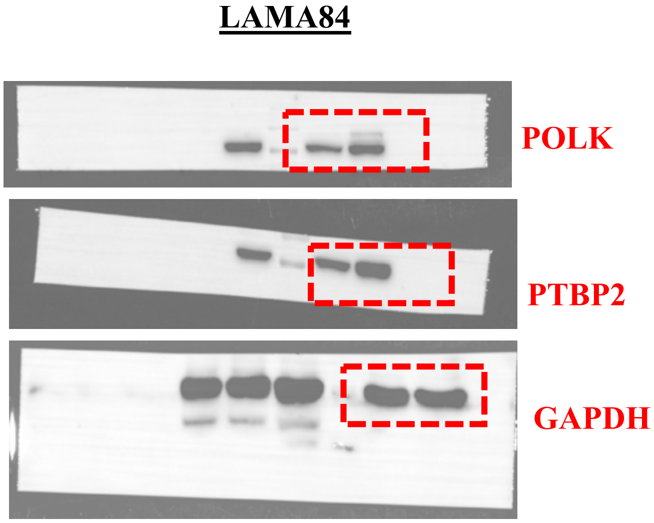

1G

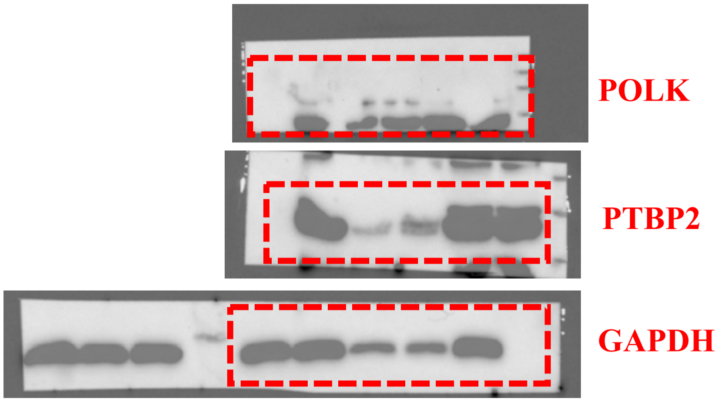

Supplementary Fig.2

Uncropped & unedited western blots

2B

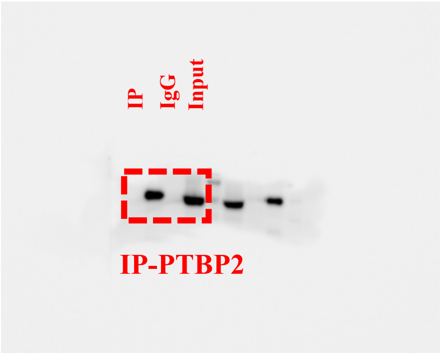

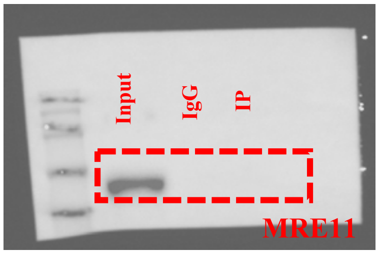

Fig.3

Uncropped & unedited western blots

3B

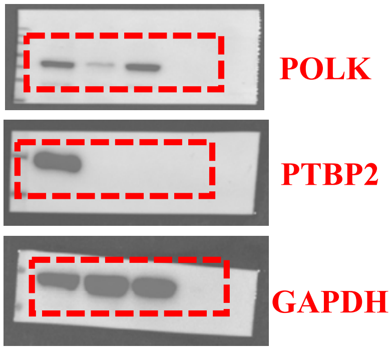

3E

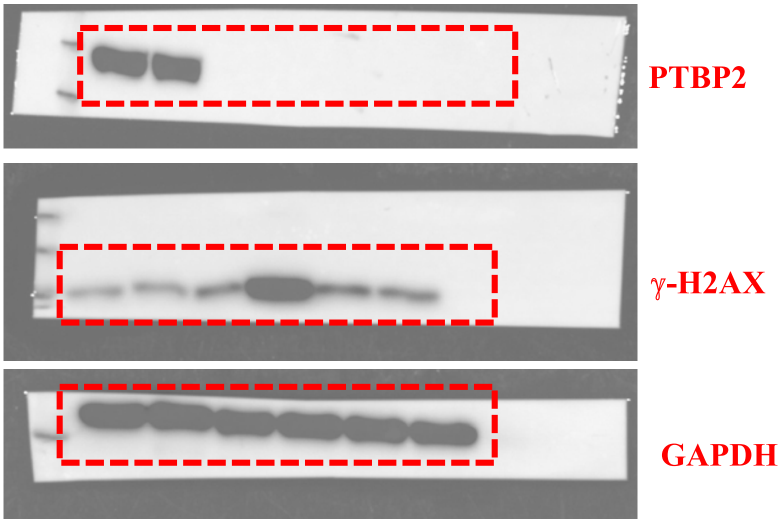

Supplementary Fig.3

Uncropped & unedited western blots

3G

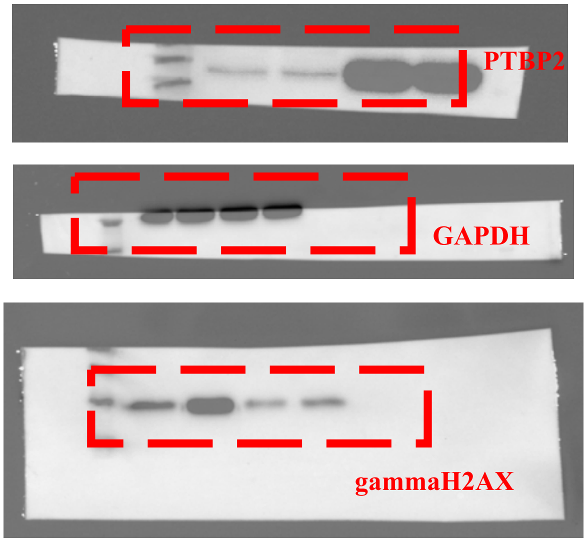

3K

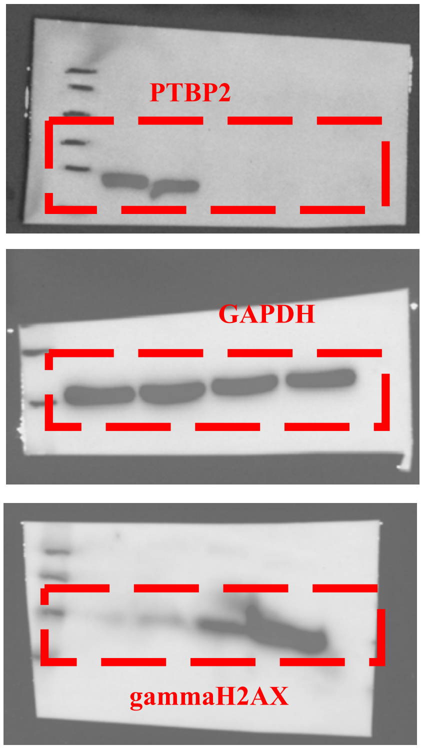

Uncropped & unedited western blots

Fig.4

4A

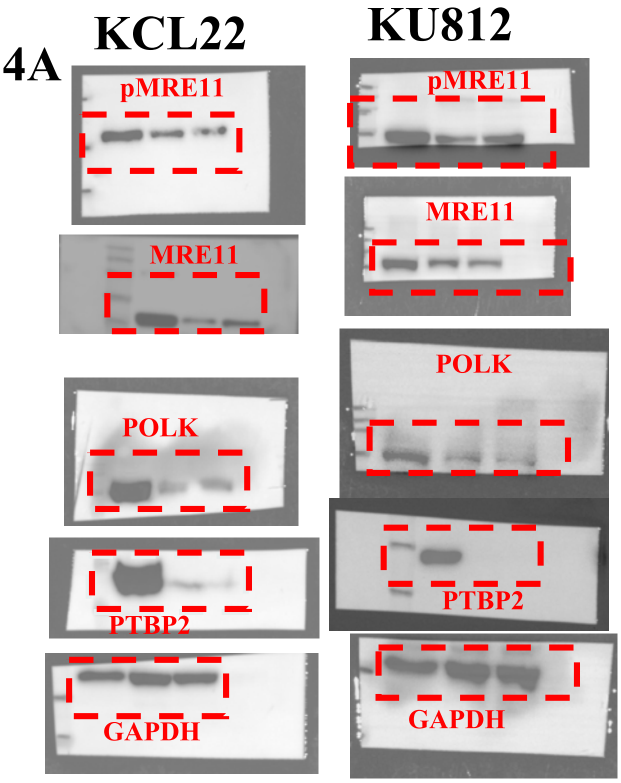

4B

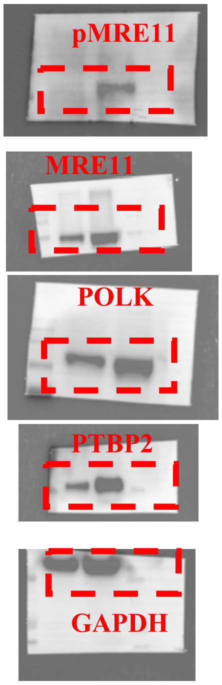

4C

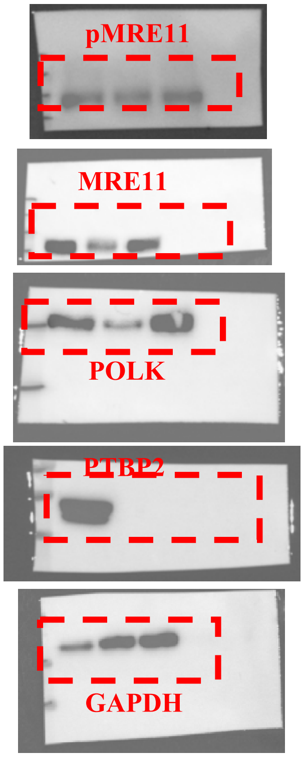

4D

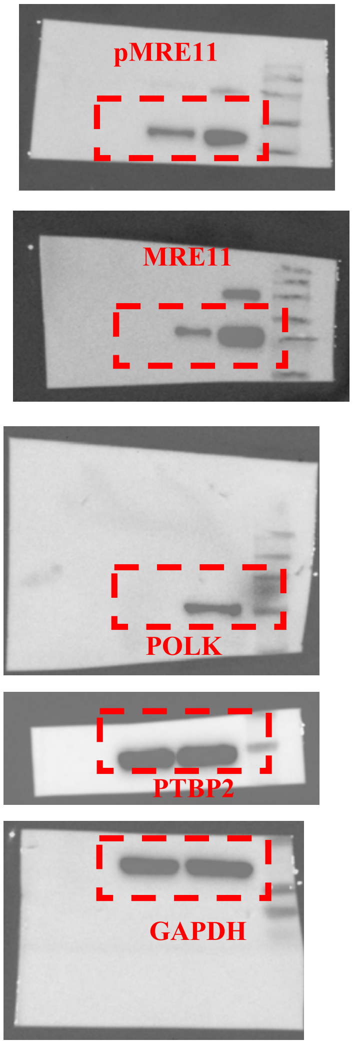

Fig.4

4E

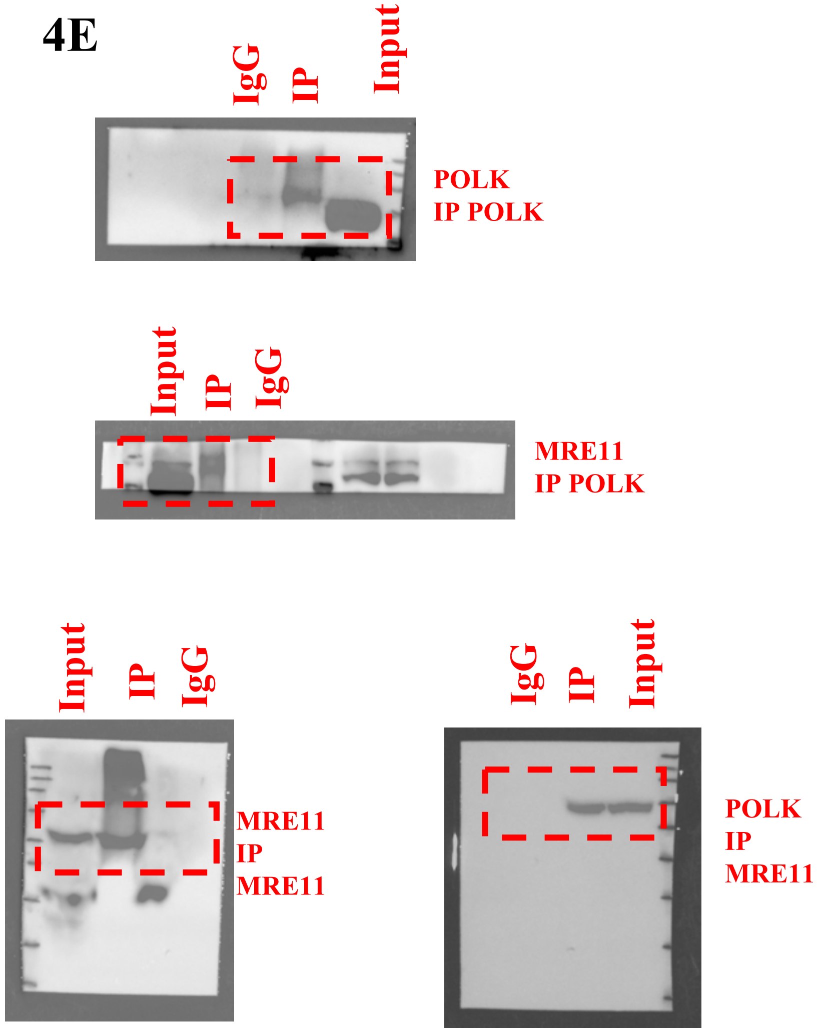
IgG
IP
Input
POLK
IP POLK
Input
IP
IgG
MRE11
IP POLK
Input
IP
IgG
MRE11
IP
MRE11
IgG
IP
Input
POLK
IP
MRE11

4G

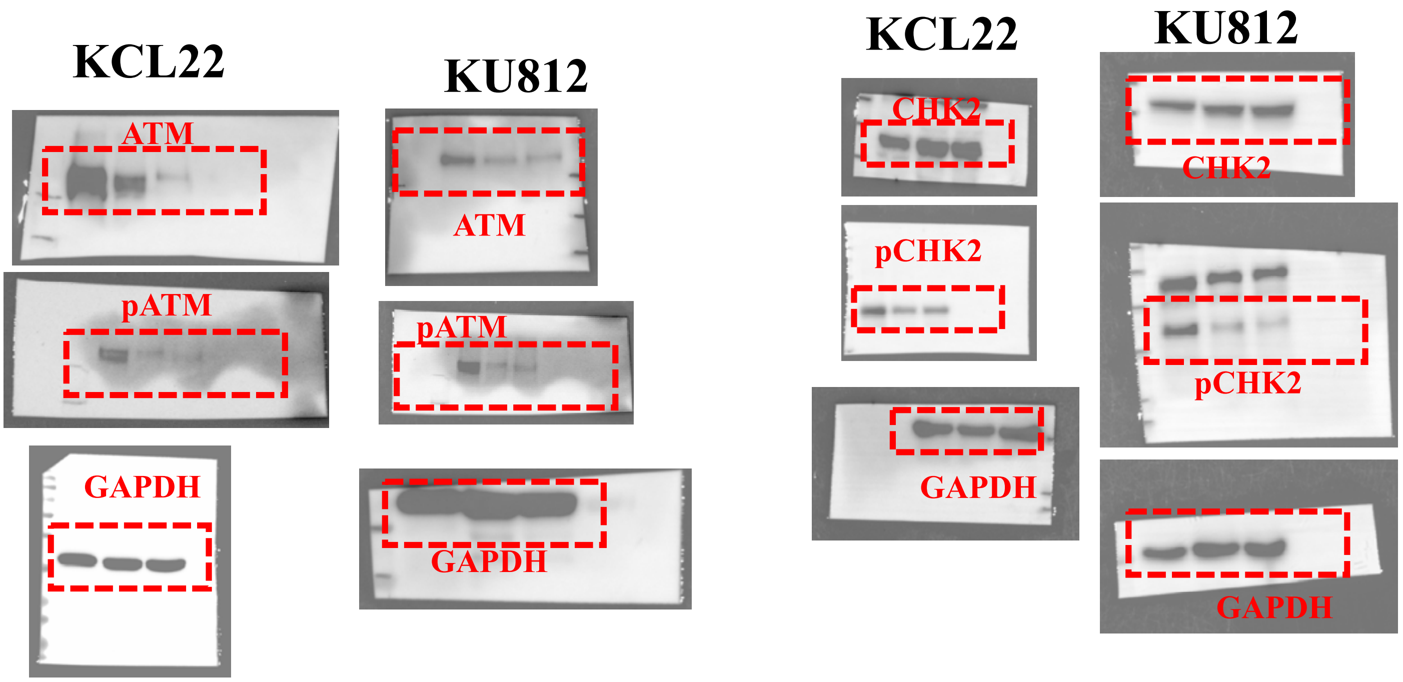
KCL22
KU812
ATM
ATM
pATM
pATM
GAPDH
GAPDH
KCL22
KU812
CHK2
CHK2
pCHK2
pCHK2
GAPDH
GAPDH

4H

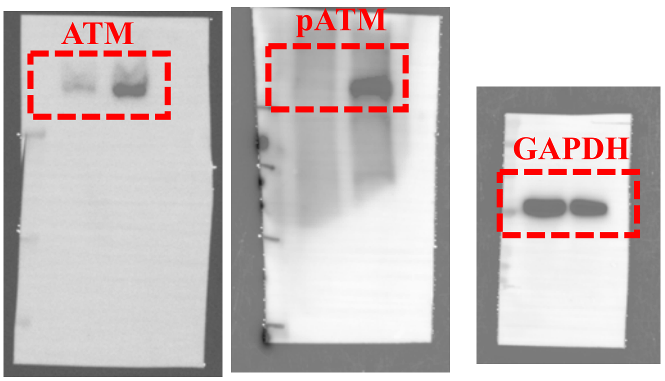
ATM
pATM
GAPDH

4G

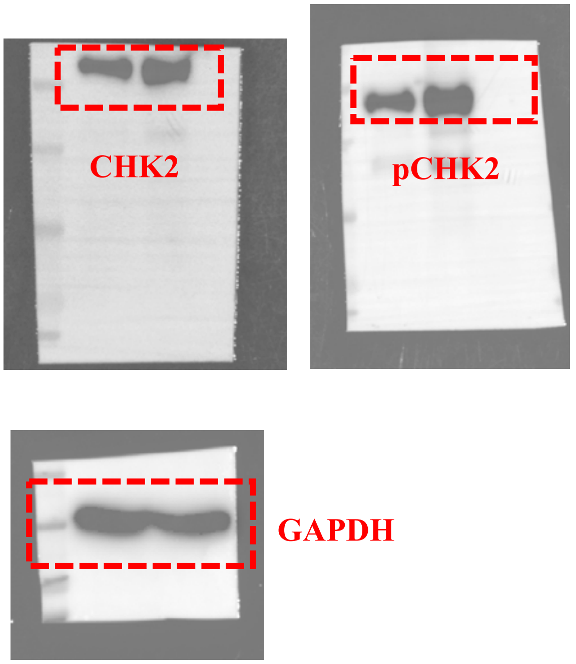
CHK2
pCHK2
GAPDH

Fig.6

Uncropped & unedited western blots

6C

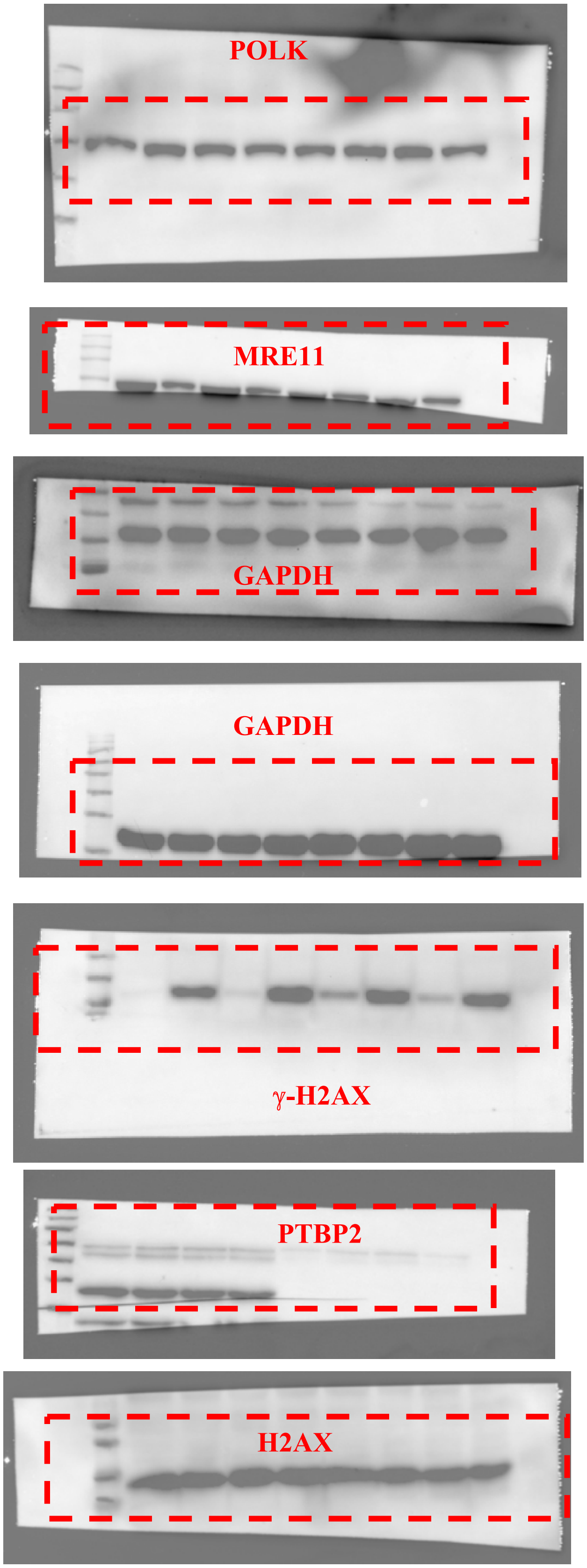

6D

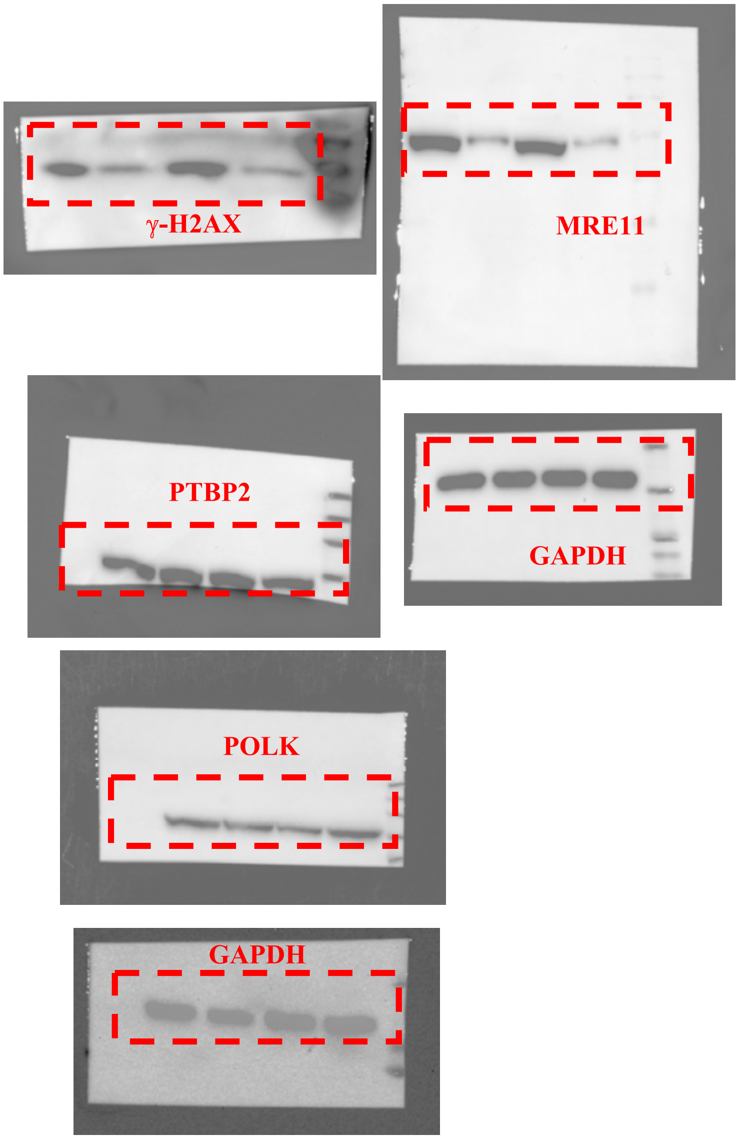

Supplementary fig 8

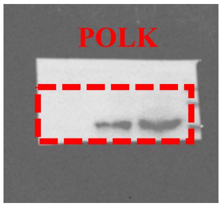

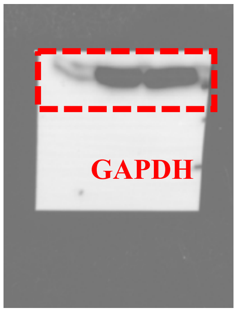

Supplement: Supplementary file 12 — Raw unedited blots [file 41420_2026_2951_MOESM12_ESM.pdf]
